# AΙ-Driven Interventions for Neurocognitive, Self-Regulation, and Adaptive Skill Development in Neurodevelopmental and Cognitive Disorders: A Systematic Review of Randomized Controlled Trials

**DOI:** 10.3390/healthcare14183102

**Published:** 2026-09-20

**Authors:** Eleni Mitsea, Athanasios Drigas, Charalabos Skianis

**Affiliations:** 1 Net Media Lab & Mind & Brain R&D, Institute of Informatics & Telecommunications, National Centre of Scientific Research ‘Demokritos’ Athens, 15341 Agia Paraskevi, Greece; dr@iit.demokritos.gr; 2 Department of Information and Communication Systems Engineering, University of Aegean, 82300 Mytilene, Greece; cskianis@aegean.gr

**Keywords:** artificial intelligence, neurodevelopmental and cognitive disorders, cognitive and executive functions, metacognition and self-regulation, adaptive skills, social-emotional skills

## Abstract

**Background**: Artificial intelligence (AI) is increasingly being used in interventions among individuals with neurodevelopmental and cognitive disorders, offering personalized and adaptive approaches that advance traditional therapeutic practices. Although previous reviews have focused on symptom detection or alleviation, less attention has been paid to the impact of AI in fostering the acquisition of higher-order skills essential for being functional and independent. This review uniquely addresses this gap by synthesizing evidence from randomized controlled trials on AI-driven skill acquisition across multiple domains. **Objectives**: The objective of this systematic review is to synthesize evidence from randomized controlled trials evaluating the effectiveness of AI-driven interventions in promoting skillfulness. More specifically, it investigates the acquisition of neurocognitive, self-regulation, and adaptive and related skills among individuals with neurodevelopmental and cognitive disorders, including attention deficit and hyperactivity disorder, autism spectrum disorder, dyslexia, dyscalculia, and cognitive impairment. **Methods**: A systematic search, according to the PRISMA 2020 guidelines, was conducted, selecting randomized controlled trials published between 2019 and 2026. Eligible technologies included conversational agents, intelligent tutoring systems, adaptive training platforms, and machine learning-based interventions. Risk of bias was assessed using the Cochrane Risk of Bias 2 tool. **Results:** Twenty-four randomized controlled trials met the inclusion criteria. The findings demonstrated improvements in a wide range of skills, such as attention, working memory, mental flexibility, metacognitive control, emotional regulation, inhibition control, and social and communication skills. Generative AI showed efficacy for language and communication skills, while machine learning-based systems demonstrated positive effects on attention regulation and self-regulation. **Conclusions**: This review concludes that artificial intelligence can effectively assist conventional interventions for individuals with neurodevelopmental and cognitive disorders. However, the heterogeneity in intervention designs, outcome measures, and participant populations limits generalizability and highlights the need for standardized assessment frameworks, larger-scale longitudinal trials, and mechanistic investigations to translate these preliminary gains into long-term functional improvements across diverse clinical and cultural contexts.

## 1. Introduction

Neurodevelopmental disorders (NDDs) refer to a set of clinical conditions that emerge early in development and persist across the lifespan, creating enduring functional challenges. Cognitive disorders refer to both acquired and developmental conditions characterized by cognitive dysfunction [1]. Neurodevelopmental disorders include, among others, attention-deficit/hyperactivity disorder (ADHD), autism spectrum disorder (ASD), specific learning disorders (SLDs) (i.e., dyslexia and dyscalculia), and developmental coordination disorder (DCDs). Although each disorder is defined by a distinct pattern of core symptoms, all of these conditions are associated with difficulties in daily living, educational attainment, and social relationships [2]. Given that these disorders endure throughout an individual’s life, interventions must target sustained improvements in daily functioning rather than providing temporary symptom relief. Thus, it is very important to target the underlying skills that people need in order to deal with real-life challenges.

Despite their diagnostic differences, these neurodevelopmental disorders converge on a set of shared neuropsychological and functional deficits. Impairments in neurocognitive functioning, including attentional processes, working memory, inhibitory control, and mental flexibility, are documented across ADHD, ASD, and specific learning disorders [3,4]. Difficulties with sustained attention, self-regulation, adaptive functioning, and academic participation are also common [1,5]. Impairments in social functioning are common across these disorders, extending beyond the main social difficulties that define ASD to disrupt peer relationships in ADHD, DCD, and other neurodevelopmental conditions [6].

This overlap suggests that targeting shared functional domains may lead to broader benefits. Because the same neurocognitive and self-regulatory difficulties underpin functional impairment across several disorders, interventions designed to train these underlying processes can improve outcomes in multiple areas, including academic performance, social interactions, and daily living skills [4].

It is important to outline that neurodevelopmental and cognitive disorders differ in etiology, onset, developmental course and clinical presentation. The present review adopts a functional rather than diagnostic perspective. The rationale for combining these populations is based on the presence of overlapping neurocognitive, self-regulatory, and adaptive difficulties that may be targeted through similar skill-oriented intervention mechanisms. This approach does not imply that these populations are clinically equivalent or that AI interventions will have comparable effects across diagnoses. Instead, it allows examination of whether AI-assisted interventions can support shared functional processes across heterogeneous populations. The findings should therefore be interpreted at the level of skill domains, as well as diagnostic groups with attention to population-specific differences.

Contemporary intervention science has shifted its emphasis from symptom reduction toward the enhancement of real-world skills and functional participation. The World Health Organization’s International Classification of Functioning, Disability, and Health (2001) [7] frames outcomes in terms of activity and participation, and this view has been adopted in neurodevelopmental care [8]. Accordingly, interventions that train neurocognitive function and self-regulation skills can improve adaptive outcomes, reflecting a priority on building competencies rather than merely suppressing symptoms [9]. This skills-oriented perspective recognizes that improved daily functioning can be a major goal of treatment.

Traditional digital interventions for neurodevelopmental disorders are typically static, rule-based, and identical for every child. A computerized training program for working memory, for example, presents the same sequence of trials regardless of the user’s ongoing performance, error patterns, or motivational state. Feedback, if present, is often generic and pre-programmed. While such tools can improve performance on the trained tasks themselves, their design limits transfer to real-world skills, especially in heterogeneous populations [4]. These systems remain passive software that cannot sense, interpret, or respond to a child’s changing needs.

Artificial intelligence can address the aforementioned limitations. AI-enabled systems can continuously analyze users’ data (i.e., response times, error types, speech patterns, or facial expressions) and adjust the intervention in real time. Personalization replaces uniformity since the AI system adapts the difficulty according to the individual’s needs. Prediction algorithms anticipate when the individual is likely to disengage or fail, triggering proactive support. Intelligent feedback becomes formative, offering hints and explanations that scaffold training. Conversational interaction through natural language allows the individual to ask questions and receive context-sensitive guidance. Real-time monitoring captures subtle behavioral markers that the human eye cannot perceive, and automated scaffolding dynamically adjusts the level of assistance to keep the individual in an optimal zone of challenge [10]. In such ways, AI can transform digital tools from passive training programs into adaptive intervention systems.

Several fields of AI underpin this transition. Intelligent tutoring systems (ITSs) use machine-learning models of the trainee to select the best line of action, a method that has been proven effective in interventions for children with dyscalculia [11]. Machine learning facilitates the identification of patterns in large datasets, thereby enhancing predictive models for treatment response [12]. Reinforcement-learning algorithms can optimize sequences of intervention tasks by maximizing engagement or skill acquisition [13]. Computer vision opens the door to analyzing motor coordination in developmental coordination disorder, as well as the tracking of facial emotion during social skills training [14,15]. Finally, large language models and generative artificial intelligence may be used to develop personalized therapeutic interventions, conversational practice partners [16], and explanations adapted to a person’s language level and interests [17]. These technologies are not yet conventional in clinical practice, but they are rapidly evolving from proof-of-concept research to scalable, evidence-based intervention platforms.

This reorientation of intervention goals toward functional skills is in line with evidence that cognitive, self-regulation, and adaptive skills are the main mechanisms by which neurodevelopmental disorders limit participation and quality of life [7]. For the purposes of this review, we define neurocognitive skills as the basic information-processing capacities that enable goal-directed behavior, including attention, working memory, inhibitory control, mental flexibility, and processing speed. Self-regulation skills encompass the metacognitive, emotional, and behavioral control processes that allow individuals to reflect on their performance, regulate their thoughts and emotions, and adjust their behavior in response to environmental demands. Adaptive skills represent the observable expression of these cognitive and self-regulatory capacities in everyday life, including social interaction, communication, daily living, and academic functioning. These domains are conceptualized as an interconnected system with neurocognitive skills providing the foundational architecture, self-regulation providing the control processes, and adaptive skills representing the real-world application of these competencies [18,19,20,21,22,23].

Rather than just being related features, these skills mediate between core deficits and everyday outcomes [18,19]. Working memory, inhibitory control, planning, attentional control, and mental flexibility support goal-directed behavior and are significant indicators of academic achievement and social competence [20]. Self-regulation, which consists of emotional regulation, impulse control, and behavioral inhibition, influences an individual’s capacity to manage frustration, deal with everyday demands, and sustain engagement. Self-regulation is recognized as an indicator of positive mental health and life satisfaction [19,21]. Adaptive skills, including communication skills, social interaction, independence, daily living, and academic functioning, demonstrate the effective integration of cognitive and self-regulatory capabilities into real-world performance. They are among the most significant indicators of community participation and perceived quality of life among children with neurodevelopmental conditions [22,23].

A growing body of systematic reviews has examined the role of artificial intelligence in neurodevelopmental disorders. However, the focus has remained mainly on classification, screening, and diagnosis. For instance, several syntheses have evaluated machine-learning algorithms designed to differentiate children with autism spectrum disorder from typically developing peers [24]. Other studies have reviewed AI-based approaches for identifying attention-deficit/hyperactivity disorder through neuropsychological test scores, electroencephalography, and structural brain imaging [25]. Reviews in the domain of specific learning disorders have similarly emphasized the detection of dyslexia and dyscalculia utilizing automated linguistic or mathematical feature analysis [26]. More general overviews of artificial intelligence in special education have documented software tools and platforms, but without isolating randomized controlled trials or clinical populations [27]. A smaller number of reviews have examined AI-driven tools used in therapeutic settings for neurodevelopmental disorders, such as robotic platforms for ASD [28]. However, these syntheses combined randomized and non-randomized designs or were limited to a single diagnostic group. As a result, no systematic review has focused exclusively on randomized controlled trials of AI-assisted interventions for skill development in neurodevelopmental disorders. The present review addresses this gap by presenting a synthesis of such trials, mapping the evidence into skills domains.

Cognitive, self-regulatory, and adaptive skills are interdependent (i.e., improved inhibitory control supports emotion regulation, which in turn facilitates social participation). Thus, an intervention paradigm that emphasizes these overlapping competencies captures the functional architecture of adaptive living. Therefore, this review adopts this integrated paradigm to examine how AI-based interventions target these foundational skills to enhance real-world outcomes.

Thus, the literature has not been synthesized in a unified way, meeting four basic requirements: inclusion of major neurodevelopmental and cognitive disorders; restriction to randomized controlled trials; focus on AI-assisted interventions; and organization of outcomes according to the specific cognitive, self-regulatory, and adaptive skills.

This review addresses this gap by providing a synthesis of randomized controlled trials of AI-based interventions targeting foundational skills among people with neurodevelopmental and cognitive disorders.

The review examines the following research question: what is the efficacy of AI-assisted interventions on cognitive, self-regulation, and adaptive skills among people with cognitive and neurodevelopmental disorders? Taking into account the adaptive and personalized nature of artificial intelligence, it is hypothesized that AI-assisted interventions will improve participants’ skills, especially in domains that ensure self-regulated and adaptive functioning. This review aspires to contribute to the discussion about the role of artificial intelligence in promoting skill acquisition for individuals with neurodevelopmental and cognitive disorders.

## 2. Theoretical Background

### 2.1. A Conceptual Framework of Functional Skills in Neurodevelopmental and Cognitive Disorders

Functional skills in individuals with neurodevelopmental disorders depend on interconnected domains, including cognitive skills, self-regulation skills, and adaptive skills (Table 1). Cognitive skills (i.e., attention, working memory, and mental flexibility) provide the basic information-processing architecture that enables goal-directed behavior [18,29]. These foundational capacities allow an individual to hold instructions in mind, switch between tasks, and organize actions toward a goal. The research has confirmed that deficits in cognition are common among people with ADHD, ASD, and cognitive disorders, reducing their efficiency to adapt to environmental demands [4,20].

Self-regulation depends on both cognitive resources and control processes. Metacognition, which is a core component of self-regulation, allows people to reflect on their own performance and consciously regulate their thoughts, emotions, and actions when difficulties arise [30,31]. Emotional regulation and impulse control help an individual to manage frustration, delay gratification, and persist in challenging situations [19,21]. Self-regulation integrates cognitive resources with motivational and emotional processes to sustain effortful, goal-directed action over time. A recent meta-analysis demonstrated that self-regulation is a powerful predictor of mental health, academic success, and life satisfaction [19]. This domain is particularly vulnerable in neurodevelopmental disorders, where even well-developed cognitive skills may fail to translate into adaptive behavior if self-regulation skills are underdeveloped (Table 1).

Adaptive skills represent the expression of cognitive and self-regulatory capacities in everyday life. Positive and functional communication, social interaction, daily living routines, academic participation, motor coordination, all require the effective coordination of underlying cognitive and self-regulatory processes [1,32]. Adaptive behavior is defined as the set of social, emotional, and practical skills that are learned and performed to meet the demands of daily living, and it is a core component of the diagnostic criteria for intellectual disability and a key predictor of quality of life in all neurodevelopmental conditions [33] (Table 1). When cognitive skills support accurate information processing and self-regulation provides the control needed to apply those skills flexibly, people can more effectively participate in school, in work, in peer relationships, and in community settings. The ultimate aim of an intervention should be the training of the integrated system of cognitive, self-regulatory, and adaptive competencies that underpin functional participation and quality of life [7,34]. Within this review, social, emotional, communication, academic, and motor outcomes are conceptualized as components of adaptive functioning because they reflect the application of cognitive and self-regulatory capacities in everyday contexts.

**Figure 1:**
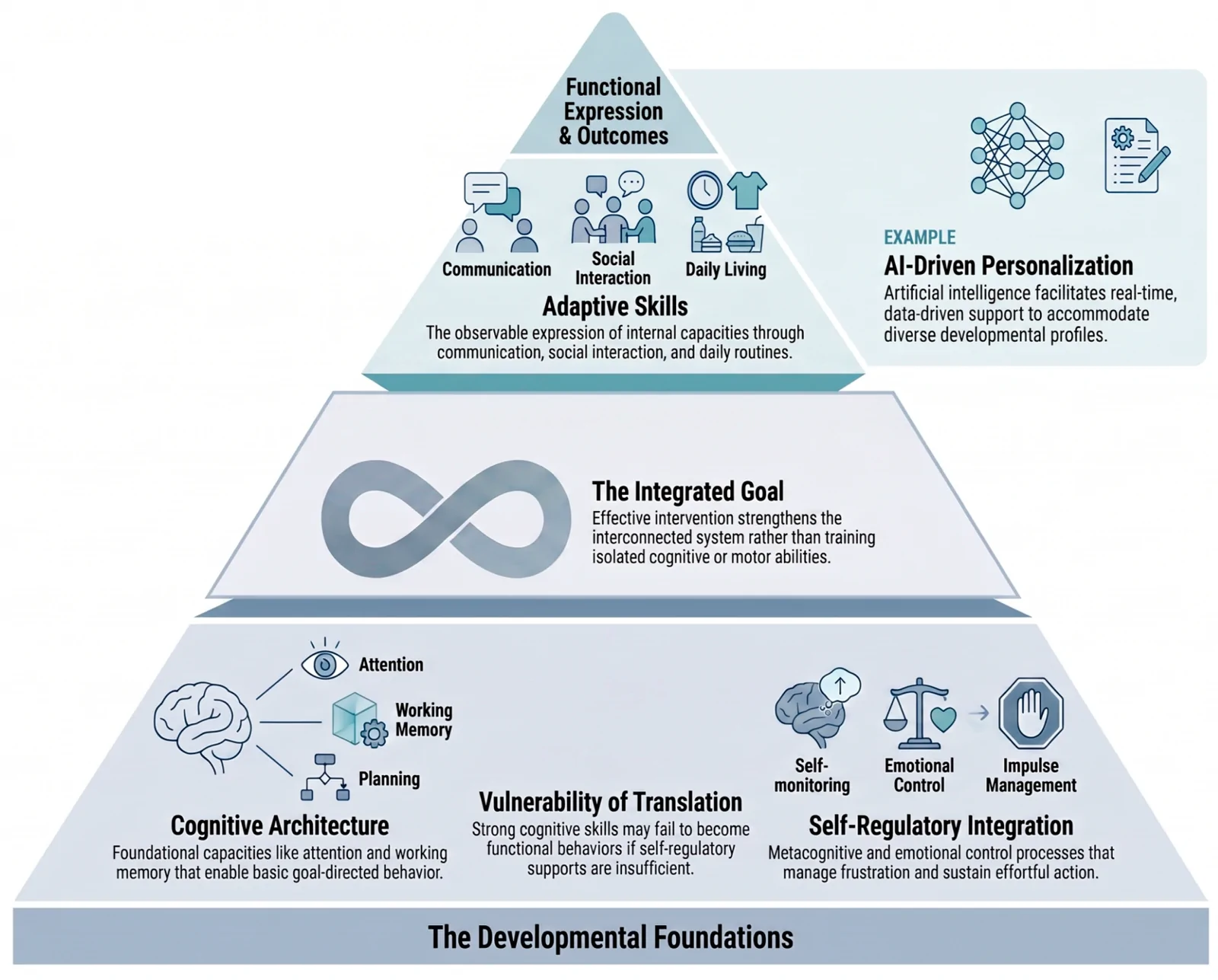
Τhe interdependence of cognitive architecture, self-regulatory integration, and adaptive skills, with AI-driven personalization supporting functional expression across communication, social interaction, and daily routines.

Given the multidimensional nature of functional skills, effective interventions must be flexible to accommodate heterogeneity in developmental profiles while providing individualized support, continuous monitoring, and adaptive guidance. Artificial intelligence offers a promising framework for operationalizing these intervention principles through data-driven personalization, real-time adaptation, and intelligent decision support [9]. As shown in the following figure, an effective intervention must improve the interconnected system of foundational cognitive capacities and self-regulatory processes, rather than training isolated abilities (Figure 1).

### 2.2. Artificial Intelligence as an Adaptive Framework for Functional Skill Development

Effective interventions for skills training, especially among people with neurodevelopmental disorders, require systematic practice, individualized instruction, continuous performance monitoring, and immediate feedback [9]. However, conventional interventions are often constrained by limited therapist availability, standard instructional protocols, and difficulties in adjusting intervention to the trainees’ changing needs [35,36]. Recent advances in artificial intelligence provide an opportunity to address these challenges by enabling adaptive, data-driven, and personalized intervention strategies that respond dynamically to individual performance and training progress [37,38].

Artificial intelligence operationalizes established intervention processes, including personalization, adaptive task progression, continuous monitoring, formative feedback, scaffolding, and predictive decision support with greater precision. Personalization and continuous adaptation ensure that task demands match the individual’s current ability, maintaining engagement and preventing boredom [37,39]. The immediate feedback provides trainees precise information about their performance, which accelerates skill development [40]. AI-based systems also allow continuous assessment that captures subtle fluctuations in performance and guides real-time adjustments according to the intervention plan [41]. Scaffolding resembles realistic coaching that children and adults with neurodevelopmental disorders need in therapeutic settings [42]. Predictive algorithms improve intervention by anticipating disengagement or regression and providing proactive help before the trainee gives up [43]. Such AI-enabled capabilities can transform conventional interventions into a continuously adaptive process that can deliver individualized support.

The adaptive capabilities of AI systems operationalize established learning theories. For instance, the real-time adjustment of task difficulty based on individual performance maintains learners within Vygotsky’s Zone of Proximal Development, ensuring that challenges are neither too easy nor too difficult [39]. Furthermore, the scaffolding provided by conversational agents and adaptive platforms supports the development of self-regulation skills by transferring responsibility from the system to the learner, consistent with Zimmerman’s model of self-regulated learning [30,31].

The AI tools have the potential to address the specific needs that characterize cognitive and neurodevelopmental disorders (Table 2). Individuals with ADHD, ASD, ID, and SLD present heterogeneous cognitive and developmental characteristics. Personalization algorithms can help by tailoring task content and difficulty to each trainee’s current ability. Evidence has shown that such approaches support skill development [11]. Performance in cognitive and neurodevelopmental disorders often fluctuates. Adaptive algorithms and reinforcement learning can adjust task demands in real time to match these variations [44]. Recent studies have demonstrated that this dynamic adaptation improves self-regulation and related self-control skills [45]. Continuous monitoring is essential because attention and engagement are not stable in these populations. Computer vision and wearable sensors, for instance, can capture subtle changes in behavior and attentional state without interrupting the intervention [46]. Many individuals with cognitive and neurodevelopmental disorders struggle to internalize delayed consequences. They need immediate and formative feedback. Intelligent tutoring systems and conversational agents can provide this feedback. Studies have shown that such systems can improve metacognitive awareness, and language and communication skills [47,48]. Systematic practice is necessary to acquire skills that peers with typical development acquire with less effort. Adaptive cognitive-training platforms and digital therapeutics can deliver this structured practice. Meta-analytic evidence indicates gains in cognitive and social domains from such interventions [49].

Finally, low motivation and poor treatment adherence are common challenges. Predictive analytics can detect early signs of disengagement and trigger proactive adjustments. This capability helps sustain persistence and supports adaptive functioning [50]. AI-driven mechanisms seem to have the potential to address the main barriers to skill acquisition in neurodevelopmental and cognitive disorders and to improve the cognitive, self-regulation, and adaptive competencies outlined in the conceptual framework.

## 3. Materials and Methods

### 3.1. Study Design

This systematic review was performed following the methodological standards outlined in the PRISMA 2020 framework [51], which offers structured recommendations for planning, identifying, screening, and integrating empirical evidence. The completed PRISMA checklist can be downloaded at Supplementary Materials. The central aim of the review was to assess whether AI-driven interventions enhance neurocognitive, self-regulatory, adaptive, and related functional skills among children, adolescents, and adults diagnosed with neurodevelopmental and cognitive disorders.

The protocol specifying the research question, inclusion and exclusion criteria, and analytical procedures was registered with the Open Science Framework (available at https://osf.io/2wrtc, accessed on 16 August 2026) [52]. The review was undertaken between February 2026 and June 2026 by two independent researchers, who evaluated titles and abstracts, screened full-text articles, and retrieved relevant information from the selected studies. Any disagreements were discussed among the reviewers and the third reviewer.

### 3.2. Eligibility Criteria

The PICOS framework was employed to define the eligibility criteria [51]. The PICOS framework consists of five key elements: (a) population, (b) intervention, (c) comparison, (d) outcomes, and (e) study design. A summary of the inclusion and exclusion criteria is shown in Table 3. More specifically, regarding population, studies involving children, adolescents, or adults diagnosed with neurodevelopmental and cognitive disorders were eligible. Studies of children identified as at risk for a neurodevelopmental disorder were eligible only when the risk was established using a validated screening instrument, the study met all inclusion criteria, and the intervention and outcome measures were in line with the review question. This decision was made to avoid excluding the small but relevant literature. Studies focusing on neurological and psychiatric disorders (i.e., depression, anxiety disorders, and schizophrenia) were excluded. As mentioned, eligible participants were classified into two broad diagnostic categories. It is important to note that both categories are broad umbrella terms that encompass a wide range of subcategories. Neurodevelopmental disorders include, among others, attention-deficit/hyperactivity disorder (ADHD), autism spectrum disorder (ASD), specific learning disorders (i.e., dyslexia and dyscalculia), intellectual disabilities, and developmental coordination disorder. Similarly, cognitive disorders encompass a heterogeneous group of acquired and developmental conditions, including mild cognitive impairment, major and mild neurocognitive disorders of various etiologies, and other conditions characterized by cognitive dysfunction. Cognitive disorders comprise a broad and heterogeneous group of conditions. In this review, this category was represented by studies of mild cognitive impairment (MCI). The decision to focus on neurodevelopmental and cognitive disorders, and within the latter, on mild cognitive impairment (MCI), was guided by the following reasons. The two categories share a common functional profile. Although neurodevelopmental and cognitive disorders differ in etiology, they converge on a set of overlapping neurocognitive, self-regulatory, and adaptive difficulties [3,4,19,23]. Because these shared processes are the targets of the AI-based interventions examined in this review, grouping the two categories allowed us to examine whether AI-supported interventions can strengthen the same underlying functional mechanisms across diagnostically different populations. Furthermore, this review adopts a functional rather than a diagnostic perspective. The World Health Organization frames outcomes in terms of activity, participation, and real-world functioning rather than diagnostic labels alone [7]. The two categories were therefore combined not because they are clinically equivalent, but because they present overlapping skill-acquisition needs that AI-based interventions are designed to address. As regards the cognitive disorders, we focused on mild cognitive disorder, because it is the cognitive disorder for which AI-based cognitive training has been frequently evaluated in randomized controlled trials, providing evidence relevant to the central research question.

We emphasize that combining these categories does not imply that they are clinically equivalent or that AI interventions will have comparable effects across diagnoses. Both categories are heterogeneous and the populations differ in etiology. For this reason, findings are reported at the level of specific populations, as well as at the level of skill domains, and all conclusions are qualified by the diagnostic context in which the evidence was generated.

Intervention: Studies evaluating artificial intelligence-based interventions designed to assist cognitive, self-regulatory, and adaptive functioning were included. Eligible interventions included AI-assisted cognitive training, intelligent tutoring systems, adaptive-learning platforms, conversational AI (i.e., chatbots and large language models), AI-supported digital therapeutics, and wearable AI technologies. Comparator: Eligible comparators included treatment as usual, conventional training or therapeutic interventions, active digital controls, waitlist controls, placebo interventions, or no intervention. Outcomes: Studies were required to report at least one quantitative outcome related to neurocognitive, self-regulation, adaptive, and related skills. Study Design: Only randomized controlled trials (RCTs) published in peer-reviewed journals were included. Conference abstracts, study protocols without outcome data, case reports, qualitative studies, observational studies, reviews, editorials, and non-peer-reviewed publications were excluded.

### 3.3. Information Sources

To identify relevant studies for this systematic review, a systematic search was conducted across the following electronic databases: PubMed, Web of Science, Scopus, PsycINFO, and Google Scholar. These databases were selected because they provide peer-reviewed studies in medicine, psychology, and computer science, ensuring access to studies examining AI-based interventions for neurodevelopmental disorders. The search was restricted to peer-reviewed journal articles published in English between January 2019 and June 2026. This timeframe was chosen to capture the rapid growth of AI applications in healthcare driven by large language models.

The search was restricted to peer-reviewed journal articles published in English between January 2019 and June 2026. This timeframe was chosen to capture the rapid growth of AI applications in healthcare driven by large language models. No other date restrictions were applied. The English-only restriction was implemented to ensure accurate interpretation of the included studies. However, we acknowledge that this introduces a potential language bias, as relevant studies published in other languages may have been excluded.

### 3.4. Search Strategy

The search strategy was developed using a combination of keywords, with relevant adaptations according to the indexing system and search syntax of each database (Table 4). Boolean operators and phrase searching were applied. Database filters were used when available to identify randomized controlled trials. The search terms were organized around four main concepts: artificial intelligence, neurodevelopmental and cognitive disorders, intervention, and randomized controlled trials.

### 3.5. Selection Process

All records retrieved from the database searches were exported to reference management software (Mendeley Reference Manager, version 1.19.8), where duplicate entries were automatically identified and removed. Following deduplication, the remaining records were screened. Two reviewers independently screened the titles and abstracts of all retrieved records according to the eligibility criteria. Studies that did not meet the inclusion criteria were excluded at this stage of processing. The full texts of all studies that passed the initial screening were retrieved and assessed in more detail. The reviewers independently evaluated each full-text article, focusing on methodological quality. Each reviewer classified studies as “include”, “exclude”, or “uncertain”. Throughout the screening process, the two reviewers met to discuss any uncertainties and resolve discrepancies. When agreement could not be reached, a third reviewer was consulted to provide the final decision. The PRISMA flow diagram was constructed to illustrate the number of records identified, screened, excluded, and included at each stage of the selection process.

### 3.6. Data Collection and Data Items Process

Data extraction was conducted independently by two reviewers, using a data extraction form developed for this review. The form was designed to capture all relevant information necessary to address the research questions and to facilitate the synthesis of findings. For each eligible study, the reviewers recorded the following information: (a) bibliographic details (author(s), publication year, and country of origin), intervention characteristics, digital design, participant characteristics (sample size, mean age, and gender), clinical diagnosis, study design, outcome measures used to assess skillfulness, and the main findings. The two reviewers independently extracted data and compared their completed forms. Any disagreements were resolved with a third reviewer. The extracted data were organized in a structured summary table (Appendix A, Table A1) to facilitate cross-study comparison and synthesis.

Data were collected according to the outcomes related to neurocognitive, self-regulatory, adaptive, and related functional skill development, as these domains were aligned with the research questions. The skills included attention, processing speed, working memory, metacognitive skills, inhibitory control, impulse control, emotional regulation, mental flexibility, social skills, language and communication skills, motor control skills, problem-solving, critical thinking, and reading skills.

The extracted outcomes were grouped into skill domains. Each reported outcome was assigned to a domain on the basis of the construct that the corresponding validated instrument was designed to measure. Where a study reported multiple outcomes within the same skill domain, it was counted only once for that domain. Where a study reported outcomes across several domains, it was counted once in each relevant domain.

It is important to mention that we did not contact authors for missing data. This decision was made because the included studies provided sufficient information for our synthesis objectives. In addition, the timeframe of the review did not allow for the iterative correspondence that would be required for obtaining missing data. However, we acknowledge that contacting authors for missing data can enhance the completeness and accuracy of the synthesized evidence, and this should be considered in future reviews.

### 3.7. Reporting Bias Assessment

The methodological quality of the included randomized controlled trials was assessed with the Cochrane Risk of Bias 2 (RoB 2) tool, which is the recommended instrument for assessing the internal validity of randomized intervention studies [53]. This tool offers a structured framework for evaluating potential sources of bias across the following domains. The first domain concerns bias arising from the randomization process. The second domain addresses bias due to deviations from intended interventions. The third domain pertains to bias due to missing outcome data. The fourth domain covers bias in measurement of the outcome. Finally, the fifth domain concerns bias in selection of the reported result. Each domain is rated as “low risk”, “some concerns”, or “high risk” based on the presence and adequacy of key methodological features.

## 4. Results

### 4.1. Study Selection and General Characteristics

**Figure 2:**
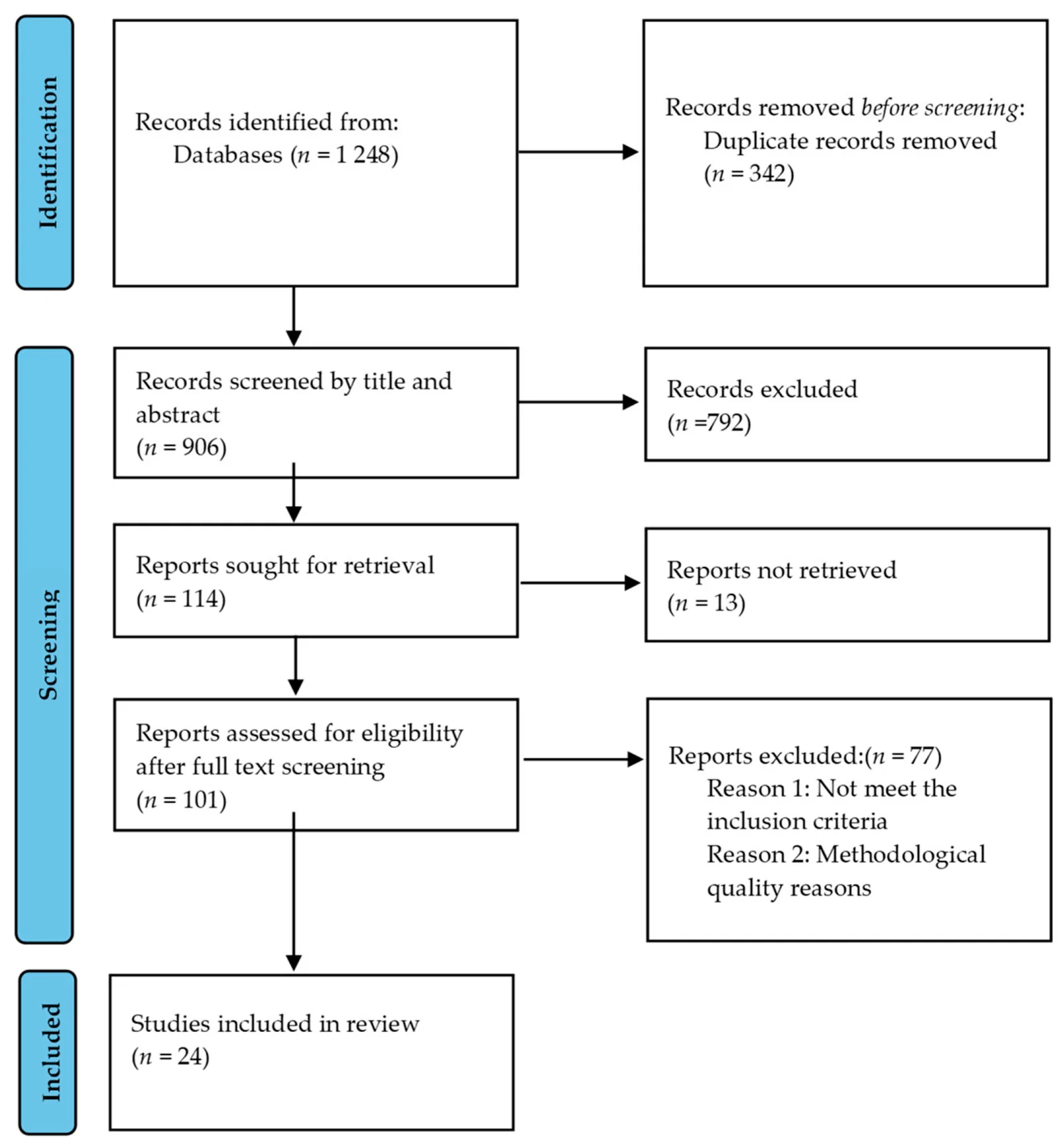
The PRISMA flow diagram.

The systematic search identified 1, 248 records. After deduplication and screening, 24 randomized controlled trials met the inclusion criteria (Figure 2). Studies were published between 2019 and 2026, with the majority (70.8%) published in 2024–2026, reflecting the growing research interest in AI-based interventions for neurodevelopmental and cognitive disorders.

Studies originated from various countries, with the highest representation from China, followed by South Korea, the USA, and Germany. Other contributing countries included Singapore, Spain, India, Saudi Arabia, Nigeria, and Turkey.

The included studies demonstrated heterogeneity in participant demographics, clinical populations, and sample sizes (Appendix A, Table A1). Most studies focused on children and adolescents between 6 and 12 years. Most study populations were male, especially in studies involving ADHD and ASD. Females were underrepresented in many studies, while two investigations included exclusively male participants. The most common intervention duration was 8 to 12 weeks. All included studies employed a randomized controlled trial design.

**Figure 3:**
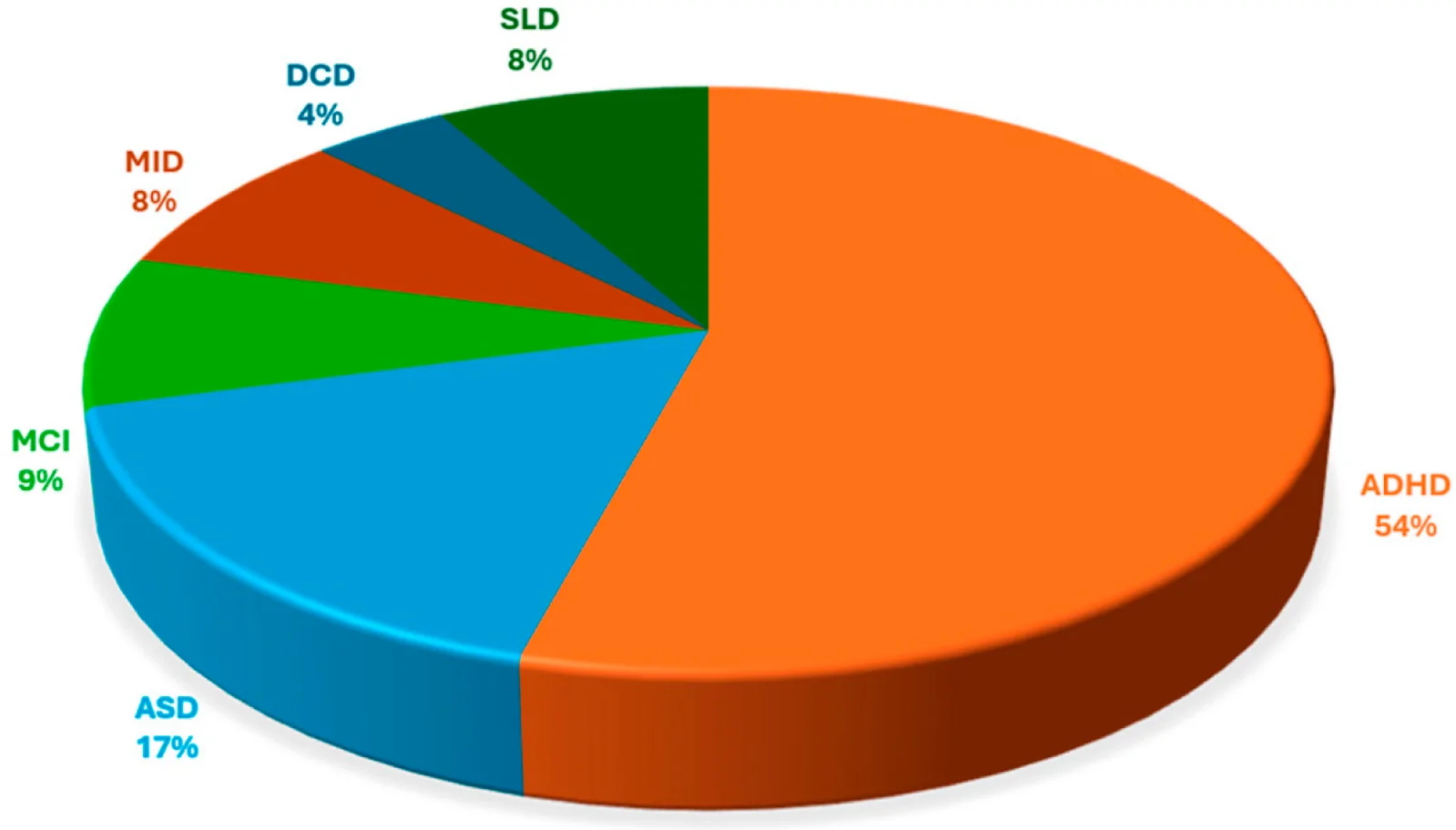
Pie chart showing the percentage of studies targeting each clinical condition across the twenty-four included randomized controlled trials.

The 24 included studies covered two broad diagnostic categories. As presented in Figure 3, among the neurodevelopmental disorders, ADHD was the most frequently investigated condition, followed by ASD; MID and SLD (including dyslexia and dyscalculia) were each represented by a smaller number of studies; and DCD was the least represented. Within the cognitive-disorders category, mild cognitive impairment was the only population represented, accounting for a modest but discernible share of the included studies. The percentages in Figure 3 represent the proportion of the 24 included studies addressing each population.

### 4.2. Characteristics of AI-Based Interventions

The selected studies employed a wide range of artificial intelligence-based interventions, including AI chatbots and conversational agents, adaptive cognitive training systems, AI-enabled wearable technologies, AI-supported educational platforms, and AI-assisted creative or therapeutic systems (Table 5). The AI tools were designed to provide personalized, adaptive, and interactive interventions by analyzing users’ performance in real time and dynamically adjusting task difficulty, feedback, or therapeutic content.

More specifically, AI chatbots and conversational agents were found to be one of the most common intervention categories. Large language model-based systems, including ChatGPT [54,55], GPT-4 [56], the Voicebot “ForMe” [57], and “Noora” [56], and chatbot-based psychoeducational platforms [58,59,60], employed natural language processing, structured dialogue, and personalized feedback to support psychoeducation, cognitive behavioral therapy, empathy training, shared reading, academic assistance, and daily routine management. These conversational AI systems were implemented in participants with attention-deficit/hyperactivity disorder, autism spectrum disorder, developmental dyslexia, and mild intellectual disability.

Adaptive cognitive training systems constituted one of the most common AI interventions. These platforms utilized machine-learning algorithms to adapt cognitive exercises in real time according to individuals’ performance and progress. Representative systems included NeuroNation MED [61,62], AKL-T01 [63], Sincrolab Digital Cognitive Therapy [64,65], Calcularis 2.0 [66], and other AI-driven cognitive games and mobile applications [67,68,69]. These interventions mainly targeted higher-order skills, such as sustained attention, inhibitory control, working memory, mental flexibility, processing speed, and academic skills (i.e., problem-solving and critical thinking), among individuals with ADHD, ASD, dyscalculia, and mild cognitive impairment.

Several studies evaluated AI-assisted wearable technologies that integrated real-time physiological or behavioral monitoring with adaptive feedback. These interventions included the Superpower Glass wearable system using Google Glass [70], wearable EEG neurofeedback devices [71], brain–computer interface-based attention training [67], and machine learning-driven neurofeedback systems [71]. By analyzing facial expressions, eye gaze, emotional responses, or neural activity, the selected AI-based interventions offered individualized feedback so as to improve attentional operations, emotion recognition, social communication, and self-regulation among children with ASD and ADHD.

AI-assisted educational platforms focused on improving academic achievement skills through adaptive and personalized training environments, such as AI tutoring systems [72], chatbot-assisted reading interventions [54,73], and AI-scaffolded educational tools [55]. The aforementioned educational platforms incorporated automated feedback, intelligent tutoring, adaptive-learning pathways, and progressive scaffolding to improve reading comprehension, vocabulary, arithmetic, language development, and problem-solving skills among children with dyslexia, ADHD, and mild intellectual disability.

Finally, AI-assisted creative and therapeutic systems integrated AI into rehabilitation and psychosocial interventions. These approaches included AI-assisted drawing therapy [74], AI-based play interventions [75], AI-supported occupational therapy [76], and AI-guided exercise rehabilitation [77]. These systems emphasized improving emotional regulation, creativity, motor coordination, and adaptive behavior, as well as functional participation. These findings demonstrated that AI interventions combining personalization, continuous performance monitoring, and real-time adaptive feedback can effectively support a wide range of skills.

**Table 5:** Classification of AI interventions included in the review.

AI- Intervention	AI-Tools/ Platforms	Clinical Populations	Target Skills	Duration	Sample Size	Selected Studies
AI Chatbots and conversational agents	ChatGPT, GPT-4, Noora, Voicebot, psychoeducation chatbots	ADHD, ASD, Dyslexia, MID	Psychoeducation, CBT, empathy, communication, reading, daily living skills	3–8 weeks	16–249	[54,55,56,57,58,59,60,73]
Adaptive cognitive training systems	AKL-T01, NeuroNation MED, Sincrolab DCT, Calcularis 2.0	ADHD, ASD, Dyscalculia, MCI	Attention, executive functions, working memory, mental flexibility	4–13 weeks	29–348	[61,62,63,64,65,66,67,68,69]
Wearable AI technologies	Superpower Glass, EEG neurofeedback, BCI systems	ASD, ADHD	Social communication, attention, emotion recognition, self-regulation	6–20 weeks	41–172	[67,70,71]
AI Educational platforms	AI tutoring systems, AI reading assistants	Dyslexia, ADHD, MID	Reading, mathematics, vocabulary, language, academic performance	5–12 weeks	70–153	[54,55,72,73]
AI creative and therapeutic systems	AI drawing therapy, AI play intervention, AI occupational therapy, AI exercise rehabilitation	ADHD, DCD	Emotional regulation, quality of life, motor skills, handwriting, adaptive functioning	4–24 weeks	41–144	[74,75,76,77]

### 4.3. Risk of Bias in the Selected Studies

**Figure 4:**
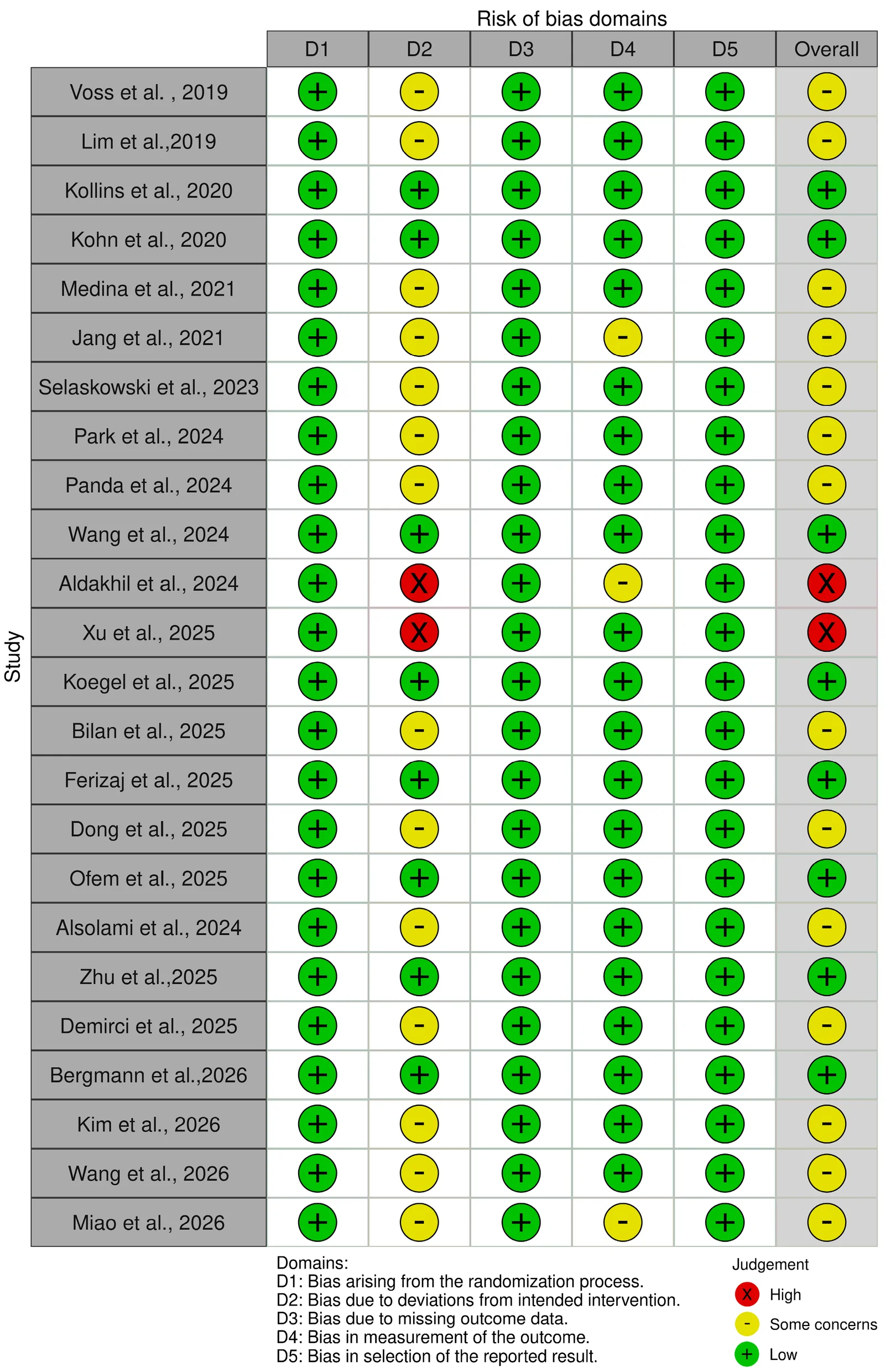
Risk-of-bias assessment of included studies using the Cochrane Risk of Bias 2 (RoB 2) tool. Green, yellow, and red symbols indicate low, some concerns, and high risk of bias, respectively, across the five domains and overall (See Refs. [54,55,56,57,58,59,60,61,62,63,64,65,66,67,68,69,70,71,72,73,74,75,76,77]).

The included studies demonstrated an acceptable level of methodological quality according to the RoB 2 criteria [53]. Most studies showed a low risk of bias arising from the randomization process. Randomization methods included computer-generated random sequences, block randomization, and concealed allocation using sequentially numbered opaque envelopes [55,63,68,69,76,77]. These procedures ensured that the baseline groups were comparable. Bias due to deviations from the intended interventions was considered a common challenge, because participants could not be blinded to the use of wearable devices or digital applications [65,75,77]. However, many studies minimized this risk by using active digital control groups [63,65] or waitlist control designs combined with careful monitoring of protocol adherence [56,61,66]. The risk of bias from missing outcome data was low. Most studies applied intention-to-treat analyses and linear mixed-effects models to account for participant attrition [63,70,71,77]. Outcome measurement bias was also minimized through the use of blinded assessors or independent psychologists who were unaware of participants’ group allocation when administering outcome measures [63]. The risk of selective reporting was reduced because the studies were registered in international trial registries [56,63,64,67,69,74,76,77]. Risk was present in designs where participants and parents could not be blinded to the intervention, thus influencing the reporting of subjective behavioral outcomes [60,75]. The overall risk-of-bias judgments for each study are summarized in Figure 4.

### 4.4. Effects of AI-Based Interventions on Skills Development

#### 4.4.1. Effects on Neurocognitive Skills

##### Attention

AI-based interventions demonstrated positive effects on attentional functioning among children with ADHD, whereas evidence in mild cognitive impairment showed no significant improvements [57,63,64,67]. More specifically, Kollins et al. [63] reported significant improvements in objective measures of attention following a digital intervention. Selaskowski et al. [59] evaluated attention outcomes in adults with ADHD following a 3-week chatbot-supported psychoeducation intervention. Both the chatbot and conventional psychoeducation app groups showed significant reductions in inattention symptoms. Similarly, Lim et al. [67] found a significant reduction in inattention scores following brain–computer interface-based training compared to waitlist control. Medina et al. [64] reported significant improvements in Conners Continuous Performance Test scores following AI-driven cognitive training. Park et al. [57] demonstrated a significant reduction in inattention symptoms as measured by the ADHD Rating Scale. Bilan et al. [65] reported significant improvements in inattentiveness scores, with an AI-driven digital cognitive therapy in children with ADHD. Xu et al. [74] evaluated an AI-assisted drawing therapy and found significant improvements in attention, with the AI group showing greater improvement than the traditional drawing group. Aldakhil [75] assessed inattention in children with ADHD following an AI-based play activities intervention and reported significant improvements in attentional functioning. However, Kim et al. [68] found no significant improvement in Digit Span Forward (DSF) following AI-driven telerehabilitation, while Ferizaj et al. [61] reported non-significant changes in attention outcomes following mobile cognitive training in adults with MCI. Bergmann et al. [62] assessed sustained attention in adults with ADHD following 12 weeks of digital cognitive training and found no significant improvements. These results indicate that AI-based interventions have the potential to improve attentional processes. However, the effectiveness may depend on the population and the type of AI-based intervention design.

##### Processing Speed

Kollins et al. [63] found improvements in reaction-time measures following an adaptive, video game-based cognitive training program delivered via iPad. Demirci et al. [76] also demonstrated significant improvements in writing speed after an AI-supported occupational therapy program that utilized touch tablets, stylus pens, and real-time AI feedback. These findings indicate that AI-based interventions can significantly enhance processing speed, with significant effects observed in motor-related processing-speed tasks.

##### Working Memory

Working-memory outcomes were assessed in seven studies showing mixed findings that appeared to be influenced by both population characteristics and intervention design. Exercise-based interventions [77] and adaptive computer-based training for dyscalculia [66] demonstrated significant improvements on complex working-memory tasks, including the Rey–Osterrieth Complex Figure Test. In contrast, two studies targeting MCI populations reported no significant working-memory gains following mobile cognitive training [61] or AI-driven telerehabilitation [68]. Medina et al. [64] demonstrated significant improvements in visuospatial working memory, as measured by the Corsi block-tapping test (backward span), following 12 weeks of AI-driven digital cognitive therapy delivered through a serious game on a mobile device in children with ADHD. On the other hand, Bergmann et al. [62] evaluated verbal working memory using the verbal learning and memory test in adults with ADHD following 12 weeks of AI-based cognitive training and found no significant improvements. Similarly, Lim et al. [67] evaluated working in children with ADHD following 8 weeks of brain–computer interface-based attention training and reported no significant improvements in working memory-related outcomes, suggesting that BCI-based training may primarily affect attention rather than working memory. These findings may indicate that working memory improvements may be task-specific and population-dependent.

##### Mental Flexibility

Four studies evaluated mental flexibility using validated neuropsychological tests, with mixed findings depending on the nature of the intervention and population. Bergmann et al. [62] found no significant improvement in mental flexibility, as measured by the TAP Flexibility task, following 12 weeks of individualized digital cognitive training with the NeuroNation MED app in adults with ADHD. In contrast, Medina et al. [64] demonstrated significant improvements in mental flexibility, as measured by the Corsi block-tapping test (backward span), following 12 weeks of AI-driven digital cognitive therapy delivered through a serious game on a mobile device. The intervention utilized a case-based reasoning algorithm that adaptively adjusted task difficulty and selection based on individual performance, suggesting that gamification and adaptive task progression may be key components for improving mental flexibility. Two studies evaluated mental flexibility using the Trail Making Test Part B, demonstrating contrasting findings regarding the role of AI. Zhu et al. [77] reported that a traditional exercise intervention, which incorporated cognitively demanding physical activity, outperformed both the AI-based intervention and the control group. The AI-driven intervention, which utilized algorithms to deliver personalized exercise prescriptions via a mobile app, also improved mental flexibility. However, its effect was smaller than the in-person supervised program. In contrast, Kim et al. [68] found no significant improvement following AI-driven telerehabilitation in adults with mild cognitive impairment. That intervention used the Zenicog platform, a self-guided, home-based cognitive training system with autonomous difficulty adjustment, but without physical activity. These findings suggest that AI can enhance mental flexibility when combined with physical activity, but passive AI-driven cognitive training alone seems insufficient.

##### Inhibitory Control

Five studies evaluated inhibitory control across diverse intervention modalities, including mHealth-guided exercise, brain–computer interfaces, and AI-supported occupational therapy. Indeed, the results showed that handwriting tasks improve inhibitory control, suggesting that interventions combining cognitive and motor demands may be particularly effective for enhancing inhibitory control. Specifically, Zhu et al. [77] found significant improvements on the Stroop Color and Word Test following both mHealth-guided and traditional exercise interventions, as compared to controls [77]. Demirci et al. (2025) [76] reported enhanced handwriting skills requiring sustained inhibitory control. Similarly, Bilan et al., Medina et al., and Lim et al. all demonstrated significant improvements on inhibition-control measures [64,65,67].

#### 4.4.2. Effects of AI-Based Interventions on Self-Regulatory Skills

##### Metacognitive-Control Skills

AI-based interventions demonstrated positive effects on higher-order capacities such as self-regulation and metacognition. Park et al. [57] reported that a voicebot-assisted intervention enhanced perceived self-regulatory capacity in children with ADHD. Participants developed greater confidence in their ability to monitor, control, and adjust their behavior during daily tasks, such as completing morning routines and homework independently. Selaskowski et al. [59] found that chatbot-assisted psychoeducation improved self-management skills in adults with ADHD, allowing participants to develop better planning, organization, and coping strategies. Beyond direct skill development, Wang et al. [73] revealed that intrinsic motivation moderated intervention effectiveness, indicating that AI interventions not only train self-regulatory skills but also foster motivational engagement. Importantly, these findings indicate that AI-based interventions operate through multiple pathways: they train self-regulatory skills, while simultaneously enhancing motivational processes.

##### Emotional Regulation

Across the selected randomized controlled trials, AI-based interventions significantly enhanced emotional regulation skills among children and adolescents with neurodevelopmental disorders. Miao et al. [60] demonstrated that AI chatbot-based dialogic reading enhanced emotional regulation among children with ADHD. The children developed better emotional control, reduced impulsive outbursts, and increased cooperative behavior during parent–child interactions. It is noteworthy that AI chatbots were as effective as professional social workers, indicating that AI can successfully teach emotional-control techniques. Jang et al. [58] reported that a mobile-based interactive chatbot delivering cognitive behavioral therapy and psychoeducation improved emotional regulation in adults with ADHD. Xu et al. [74] found that AI-assisted drawing therapy significantly reduced hyperactivity–impulsivity and oppositional defiant behavior, while enhancing emotional regulation, self-expression, and self-concept in children with ADHD. Aldakhil [75] reported that AI-based play activities that used structured gamified activities enhanced emotional well-being in children with ADHD, helping them develop emotional recognition, self-regulation, and frustration tolerance. Koegel et al. [56] found that AI-based empathy training significantly improved empathetic responses in autistic adolescents and adults, with participants learning to recognize others’ emotions, express understanding, and respond with appropriate verbal empathy. These findings demonstrate that AI-based interventions effectively develop emotional-regulation skills, including emotional control, empathy, self-regulation, and cooperative behavior.

##### Impulse Control

Impulse control outcomes were evaluated in three studies, all of which reported significant improvements in children and adults with ADHD. Both digital therapeutics [65] and exercise-based interventions [77] reduced impulsive behaviors in children with ADHD. More specifically, Bilan et al. [65] reported improvements in CPT-3 Commission scores, while Zhu et al. [77] found significant reductions in parent-rated hyperactivity/impulsivity symptoms with moderate-to-large effect sizes. It is noteworthy that mHealth-guided and traditional exercise interventions presented similar results, suggesting that AI-guided exercise delivery can be as effective as face-to-face coaching. Jang et al. [58] evaluated a mobile app-based chatbot delivering cognitive behavioral therapy and psychoeducation to adults with attention deficit. Using the CAARS Impulsivity/Emotional Lability subscale, they found a significant reduction in impulsivity symptoms following a 4-week intervention, with a large effect size (Cohen’s d = 0.83). The chatbot incorporated self-instruction training (Goal–Plan–Do–Check) and token-based economies, demonstrating that conversational AI can effectively deliver therapeutic content for impulse control.

#### 4.4.3. Effects of AI-Based Interventions on Adaptive and Quality of Life Skills

##### Social Skills

AI-based interventions demonstrated meaningful improvements in social skills, especially in studies targeting children with autism spectrum disorder. Voss et al. [70] found that a wearable AI system enhanced adaptive social behavior, as measured by the Vineland Adaptive Behavior Scales. Similarly, the study conducted by Panda et al. [69] revealed that 75% of parents observed gains in their children’s social abilities following an AI-based mobile application intervention. Similar improvements were observed among people with ADHD. Aldakhil [75] confirmed these findings, showing improvements in social functioning through AI-based play activities. As shown in the selected studies, the participants improved skills such as social initiation, enhanced peer interaction, improved emotional recognition, and adaptive social behavior. The common characteristic across these interventions was their ability to provide structured, engaging, and interactive social practice in safe and controlled environments. It is noteworthy that wearable AI systems offered real-time social feedback, mobile applications delivered structured social skills training, and play-based activities created naturalistic social opportunities. The aforementioned findings may indicate that such interventions may be especially beneficial for populations with deficits in social skills.

##### Language and Communication Skills

Evidence from five studies demonstrated that AI-based interventions have a positive impact on language and communication skills. Dong et al. [54] found that AI chatbot-led shared book reading, which utilized ChatGPT-4 with emotion-neutral feedback and dynamic pacing adjustment, improved expressive vocabulary and character reading in kindergarteners with ADHD compared to parent-led reading. A wearable EEG neurofeedback powered by machine-learning algorithms improved expressive language and sensory–cognitive awareness in children with ASD, highlighting the potential of brain–computer interfaces in language development [71]. Panda et al. [69] evaluated an AI-driven ABA-based intervention among children with ASD and found that 81% of caregivers reported improvements in their child’s verbal skills following 12 weeks of add-on treatment. Voss et al. [70] assessed the Superpower Glass wearable AI system, which provided real-time facial-expression recognition and emotion feedback in children with ASD, and the researchers reported significant improvements in social communication skills, as measured by the Vineland Adaptive Behavior Scales. Aldakhil [75] investigated an AI-based play activities intervention in children with ADHD and found significant improvements in social communication and peer interaction. The selected studies reveal that AI is particularly effective for structured language skills because it provides repetition and scaffolded practice. The findings also highlight the complementary roles of artificial intelligence and human interaction in language development. Artificial intelligence offers scalability, while humans provide emotional richness. These findings have significant practical implications for educational and clinical settings, particularly in contexts where access to specialized language instruction is limited.

##### Motor Control Skills

Two studies assessed motor control skills. AI-supported occupational therapy produced very large improvements in handwriting [76]. Exercise-based interventions significantly improved gross and fine motor skills [77].

##### Problem-Solving, Critical Thinking, and Reading Skills

AI chatbot-assisted interventions improved arithmetic word-problem-solving in students with dyslexia [73], while AI-based personalized training enhanced mathematics achievement in students with mild intellectual disabilities [72]. Adaptive computer-based training improved numerical cognition and arithmetic fluency in children with dyscalculia [66]. These findings indicate that AI-based interventions can improve problem-solving skills across diverse populations, including children with dyslexia, dyscalculia, and intellectual disabilities.

AI-based scaffolding supported higher-order academic tasks demanding critical thinking and problem-solving. Ofem et al. [55] found that AI-scaffolded planning tools helped students with mild intellectual disability to develop research skills. The intervention combined multiple AI tools for brainstorming and idea generation, literature exploration, feasibility analysis, and mapping connections between research ideas. Through step-by-step prompts, examples, and constructive feedback, these tools enabled students with cognitive impairments to tackle complex academic tasks. This finding demonstrates that AI can effectively bridge the gap between students’ current abilities and the demands of higher-order academic tasks. The success of this intervention highlights the potential of AI to support not only basic skill acquisition but also complex cognitive processes essential for academic and professional advancement, with implications for inclusive education.

##### Reading Skills

Reading skills also improved across diverse populations and technological formats. Alsolami [72] documented gains in reading achievement among students with mild intellectual disability after personalized training that leveraged multiple AI-powered educational applications with adaptive algorithms and gamification. Dong et al. [54] reported that AI chatbot-based shared book reading, delivered through ChatGPT-4 with emotion-neutral feedback and dynamic pacing, improved reading in kindergarteners with ADHD. These studies suggest that AI is especially beneficial for the development of literacy skills.

**Figure 5:**
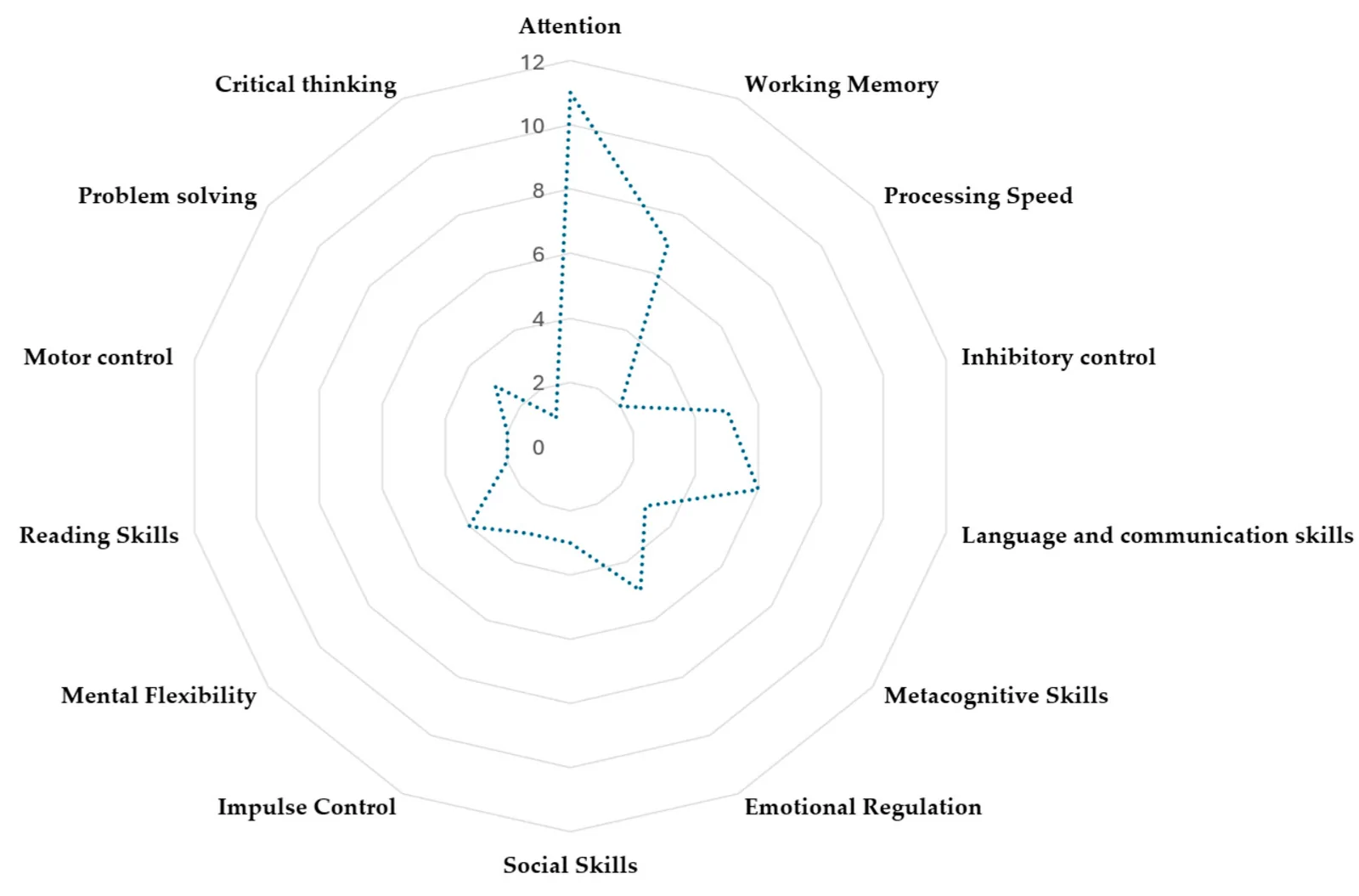
The main skills trained among the selected studies.

The distribution of AI-based intervention effects across skill domains and clinical conditions revealed distinct patterns. Attention was the skill with the most significant improvement. Among children with ADHD, the selected studies demonstrated significant enhancement in attentional functioning using diverse AI modalities, including digital therapeutics [63], brain–computer interface-based training [67], AI-driven cognitive training [64], voicebot-assisted interventions [57], and chatbot-based shared book reading [54]. Two studies targeting adults with mild cognitive impairment reported no significant attention improvements following mobile cognitive training [61] or AI-driven telerehabilitation [68]. Inhibitory control was a highly studied domain, with studies demonstrating improvements across ADHD and developmental coordination disorder [64,65,67,76,77]. Language and communication skills were also enhanced in children with ADHD [54] and autism spectrum disorder [56,71], highlighting AI’s effectiveness in training language skills because of repetition and scaffolded practice. Working-memory outcomes were assessed in seven studies with exercise-based interventions [77] and adaptive computer-based training for dyscalculia [66], for instance, demonstrating significant improvements, while studies targeting mild cognitive impairment reported no significant gains [61,68]. Improvements were observed in arithmetic word-problem-solving among students with dyslexia [73], mathematics achievement in mild intellectual disability [72], numerical cognition in dyscalculia [66], and research skills in cognitive impairment [55]. Three studies reported enhancements in metacognition and self-regulation in ADHD [57,59,73], emotional regulation in ADHD and ASD [56,60,75], and social skills in ASD [69,70,75]. Mental flexibility was assessed in four studies [62,64,68,77], and impulse control in three [58,65,77]. Processing speed, motor skills, and reading skills were each assessed in two studies [54,63,72,76,77]. The results indicate that attention, inhibitory control, and language and communication skills showed greater improvements, whereas mental flexibility and processing speed require further investigation, particularly in underrepresented populations such as MCI and DCD. Table 6 and Figure 5 present the summary of the main skills developed among different populations. Table 6 presents the studies contributing evidence to each skill domain within each population. A study reporting multiple outcomes within the same domain is listed only once for that domain, whereas a study reporting outcomes across several domains is listed once in each relevant domain.

### 4.5. Summary of Effect Sizes

It is important to mention that effect sizes were reported using different indices and comparison across studies is difficult. Effect sizes varied across skill domains and intervention types (Table 7). Large effect sizes (η^2^ ≥ 0.14; Cohen’s d ≥ 0.80) were observed for language and communication skills (η^2^ = 0.44–0.90), critical thinking (η^2^ = 0.749), and reading skills (η^2^ = 0.48–0.811). Medium to very large effects were reported for motor control (Cohen’s d = 0.48–2.291). Medium-to-large effects were found for working memory (Cohen’s d = 0.48–1.42), metacognitive skills (Cohen’s d = 0.56–0.93), inhibitory control (Cohen’s d = 0.48–0.93), impulse control (Cohen’s d = 0.60–0.83), mental flexibility (Cohen’s d = 0.63–1.19), problem-solving (η^2^ = 0.10–0.75), and emotional regulation (Cohen’s d = 0.62–0.83). Small to large effects were observed for attention (Cohen’s d = 0.28–1.08). Processing speed showed small-to-medium effects (Cohen’s d = 0.28–0.48). Social skills showed a moderate effect (Cohen’s d = 0.62). It is important to outline that the heterogeneity in effect-size reporting across studies limits direct comparisons. The absence of effect sizes in several studies [61,62,68], especially those reporting non-significant findings, should be noted as a limitation.

## 5. Discussion

### 5.1. Main Findings

The current systematic review examined the effects of AI-based interventions on skills acquisition among individuals with neurodevelopmental and cognitive disorders, focusing on neurocognitive skills, self-regulation, and adaptation skills, including social, communication, and academic domains. Analysis of twenty-four randomized controlled trials revealed that AI-based interventions demonstrated positive effects across multiple skill domains.

Cognitive training interventions using AI increased inhibitory control, working memory, and mental flexibility [64,65,77]. Individuals with ADHD were found to better regulate attention and impulsivity [57,63,67]. Social communication skills in autism spectrum disorder were enhanced with comparable gains to conventional behavioral interventions [56,69,70]. Self-regulation skills, including emotional regulation [60,75] and impulse control [65,77], improved across multiple studies. Significant improvements were observed in reading comprehension [54,72] and arithmetic word-problem-solving [66,73].

The findings of this systematic review align with several conclusions derived from earlier studies on technology-based interventions for neurodevelopmental and cognitive disorders. Cortese et al. [78], for instance, reported that digital cognitive training programs were effective for people with ADHD. The present review demonstrated that AI-based interventions can enhance specific skills, such as attentional functioning and inhibitory control, among children with ADHD [57,63,67]. Similarly, in line with Grynszpan et al. [79], who found that technology-based interventions can effectively support people diagnosed with autism spectrum disorder, the present review identified gains in social communication and adaptive social behavior skills [70,71].

The present review aspires to extend the existing literature on artificial intelligence interventions for neurodevelopmental disorders. Previous systematic reviews have advanced the field by mapping AI applications across neurodevelopmental conditions, including diagnostic systems, assistive technologies, AI educational robotics, virtual reality, and digital health solutions. However, many of these reviews focused on the technological characteristics of AI systems, the diagnostic accuracy, or the feasibility [80,81,82,83]. The present review emphasized skills acquisition and selected randomized controlled trials.

The findings also provide insights into the mechanisms through which AI may enhance intervention outcomes. Across the included RCTs, the most effective interventions incorporated adaptive learning, personalized feedback, continuous performance monitoring, and dynamic adjustment of task difficulty. The adaptive capabilities were found to enhance engagement, motivate repeated practice, and promote self-monitoring. Such characteristics distinguish AI-powered interventions from conventional digital technologies. Because several interventions combined AI components with established approaches, such as ABA, exercise, occupational therapy, or parent involvement, the observed benefits cannot be attributed to AI alone. They may reflect the combined effect of AI-supported delivery and the co-occurring therapeutic components [80].

It is important to note that the diversity of AI tools complicated the synthesis of results. Programs ranged from simple gamified apps to complex neural interfaces. Direct comparisons were challenging due to this heterogeneity. Most studies lacked long-term monitoring. Thus, it is unclear whether acquired skills can persist. AI can serve as a complementary tool to standard clinical care. It is undeniable that professional supervision is necessary to ensure safety and therapeutic alignment.

### 5.2. Clinical Implications

AI tools have significant potential to serve as assistive tools to conventional interventions. For instance, AI-based cognitive training and behavioral interventions may help people with ADHD to address core symptoms and deficits in cognition and self-regulation [57,63,67]. Digital therapeutics and chatbot-delivered cognitive behavioral interventions have demonstrated effectiveness in reducing inattention, impulsivity, and improving self-regulation [58,59]. For ASD, AI interventions targeting social communication and emotion recognition may supplement behavioral therapies, providing additional practice opportunities [56,69,70]. Wearable AI systems and chatbot-based empathy training have shown promise in enhancing social engagement and emotional understanding [56,71]. AI-assisted reading and mathematics interventions may offer personalized, multisensory support that can address the cognitive challenges which are common in dyslexia and dyscalculia [66,73]. The adaptive nature of AI-driven interventions may help maintain optimal challenge levels and promote participants’ engagement [54,72].

Clinicians and educators should consider integrating AI-driven applications, such as chatbots, adaptive-learning platforms, and wearable neurofeedback systems, into conventional intervention frameworks [64,65,76]. These tools may promote home-based practice and self-guided skill development [60,75,77]. By leveraging the scalability, accessibility, and personalization capabilities of AI, these interventions may reduce barriers to care, including geographical distance, limited specialist availability, and high treatment costs [56,72]. However, AI tools should complement rather than replace human expertise [59,62].

Beyond the immediate clinical implications, the translation of AI-based interventions into routine practice faces significant implementation challenges that have been documented across technology-driven health interventions. These include persistent issues with data standardization, clinician training gaps and the absence of reimbursement policies that would support the integration of AI tools into existing clinical workflows. The heterogeneity we observed in intervention designs and outcome measures reflects these broader challenges, as the field lacks standardized frameworks that would facilitate comparison and synthesis across studies. Furthermore, the scalability of AI interventions depends on addressing these implementation barriers, including the development of user-friendly interfaces; the establishment of clear reimbursement pathways; and the creation of data infrastructure that supports personalized prediction, adaptive feedback, continuous monitoring, and quality improvement. Future research should not only evaluate efficacy but also systematically investigate implementation strategies that can bridge the gap between evidence and practice [84,85,86].

### 5.3. Limitations

Several limitations of this systematic review are acknowledged. Although the search strategy covered five major databases, it is possible that relevant studies were missed. The restriction to English-language publications may have introduced language bias, potentially excluding valuable research published in other languages. This limitation should be considered when interpreting the generalizability of our findings, as the evidence base may be disproportionately representative of research from English-speaking contexts.

In addition, one included study [76] enrolled children identified as at risk for DCD using the Developmental Coordination Disorder Questionnaire rather than with a confirmed diagnosis. Although the risk status was established using a validated screening instrument and the study otherwise met all inclusion criteria, this difference in diagnostic certainty may affect the certainty of the conclusions drawn for the DCD population.

The heterogeneity observed across studies in terms of intervention types, AI modalities, outcome measures, and participant populations limited the feasibility of conducting quantitative meta-analyses.

Although all included studies used randomized designs, randomization alone does not guarantee high-quality or high-certainty evidence. The risk-of-bias assessment identified concerns in several domains, and the heterogeneity of interventions, outcomes, and populations further limits the certainty of the synthesized evidence The duration of interventions varied, and long-term follow-up data were often unavailable. Gender bias was a major concern, as male subjects dominated the research pool [70,72,77]. Blinding remained difficult in digital trials because participants recognized the use of specialized technology [74]. The absence of standardized outcome measures across studies limited synthesis and cross-study comparisons.

The included studies varied in their definitions and measures of adaptive skills, with some studies focusing on proximal outcomes, while others measured far transfer to real-world functioning. This variability limits our ability to draw firm conclusions about the generalizability of AI-based interventions to real-world adaptive functioning. Future research should prioritize the use of validated measures of far transfer and should explicitly distinguish between proximal training effects and meaningful functional improvements.

It is important to mention that neurodevelopmental and cognitive disorders differ in etiology, developmental trajectory, symptom profile, and intervention needs. Consequently, evidence concerning a specific skill domain in one diagnostic population cannot necessarily be generalized to another population. The present synthesis should be interpreted as a mapping of potentially shared intervention targets rather than evidence of equivalent treatment effects across diagnostic categories.

### 5.4. Future Directions

Future research should address several important questions to advance the field of AI-based interventions for neurodevelopmental and neurocognitive disorders. Large-scale randomized controlled trials with extended follow-up periods are needed to assess the durability of treatment gains [63,68,75]. Research exploring individual differences in treatment response would support personalized intervention approaches by further examining the demographic characteristics, clinical profiles, and cognitive abilities [66,73,77]. Comparative effectiveness studies between AI interventions and standard treatments, as well as between different AI modalities, would facilitate treatment selection [59,64]. Research in diverse populations would improve generalizability and inform implementation strategies [55]. Mechanistic studies investigating neurophysiological underpinnings would enhance understanding of how AI interventions promote skillfulness [65,71].

Another priority is the development of standardized core outcome sets and minimum reporting standards for AI-based interventions. Future studies should report the target skill domain, operational definition of the outcome, validated measurement instrument, intervention dose and duration, AI functionality, comparator characteristics, sample size, attrition, follow-up duration, and between-group effect estimates with measures of uncertainty. Whenever possible, studies should distinguish performance on trained tasks from transfer to untrained tasks and real-world functioning. Establishing such reporting standards would improve cross-study comparability, facilitate quantitative synthesis, and support the development of clinically meaningful evidence for AI-assisted skill development.

## 6. Conclusions

This systematic review demonstrated that AI-based interventions effectively improve neurocognitive, self-regulatory, adaptive, and related skills in individuals with neurodevelopmental and cognitive disorders. The most significant effects were observed for attention control, working memory, mental flexibility, inhibitory control, emotional regulation, social skills, and academic skills (i.e., problem-solving).

While AI interventions showed promise, several methodological limitations need careful interpretation. The heterogeneity in intervention types, outcome measures, and participant populations complicated comparisons and synthesis of findings.

The findings suggest that AI-based interventions may offer valuable options for individuals with limited access to specialized services. However, further research is needed to identify the optimal intervention parameters. The integration of AI tools in recognized intervention approaches may be the optimal option to equip people with the skills needed to be autonomous, self-regulated and adaptive in different circumstances.

The included studies suggest that AI tools may be effective for structured skill development and repetitive practice. Some findings suggest that human-delivered interaction may offer advantages for complex social learning and emotional support. However, this possibility requires dedicated comparative research. Based on these observed patterns, we hypothesize that a hybrid model, combining AI-assisted structured practice with human guidance and support, may represent an optimal approach. However, we acknowledge that this hypothesis is not supported by comparative evidence, as few included studies contrasted AI-only versus AI-plus-human interventions. Future research should investigate, in more depth, this question to determine the optimal balance and integration of AI and human components in intervention programs. Across the included studies, few adverse events were reported, and none of the reviewed trials reported serious intervention-related adverse events. However, because safety was not a pre-specified outcome in this review and was not systematically assessed in most included studies, no conclusions about the safety of AI-based interventions can be drawn. Future research should continue to refine AI-based interventions, evaluate their clinical utility, and investigate the mechanisms underlying their effectiveness.

## Figures and Tables

**Table 1:** The main skills that are commonly impaired among individuals with neurodevelopmental and cognitive disorders [19,21,23].

Skill Domain	Definition	Key Components	Clinical Vulnerability
Neurocognitive skills	Basic information-processing architecture that drives goal-directed behavior	Attention, working memory, inhibitory control, mental flexibility, and processing speed	Deficits common across ADHD, ASD, and learning disorders; reduce efficiency of learning and adaptation
Self-regulation skills	Metacognitive, emotional, and behavioral control processes that allow for reflection, regulation, and adjustment	Self-monitoring, emotional regulation, impulse control, and behavioral inhibition	Vulnerable domain in neurodevelopmental disorders; predictor of mental health, academic success, and life satisfaction
Adaptive skills	Observable expression of cognitive and self-regulatory capacities in everyday life	Social interaction, communication, motor coordination, participation, and independence	Core component of diagnostic criteria for intellectual disabilities (IDs)

**Table 2:** The alignment between intervention needs, evidence-based therapeutic principles, and AI capabilities. The AI capabilities address the core barriers that characterize neurodevelopmental and cognitive disorders. Consequently, AI may facilitate the simultaneous development of cognitive, self-regulation and adaptive skills through adaptive, individualized, and continuously responsive intervention [11,45,47,48,49].

Intervention Need in Neurodevelopmental and Cognitive Disorders	Core Intervention Need	AI Capability	Skill Domains Supported
Heterogeneous developmental and cognitive profiles	Individualized intervention	Personalization	Cognitive, academic, and adaptive
Fluctuating performance and developmental progression	Dynamic adaptation	Adaptive algorithms and reinforcement learning	Executive functions, metacognition, and self-regulation
Need for continuous assessment	Ongoing monitoring	Adaptive algorithms and reinforcement learning	Attention, engagement, motor coordination, and adaptive behavior
Requirement for immediate reinforcement	Formative feedback	Intelligent tutoring systems and conversational AI	Metacognition, language, communication, and self-regulation
Need for intensive and repetitive practice	Structured practice	Adaptive cognitive training and digital therapeutics	Cognitive, social, academic, and motor skills
Low motivation and commitment to therapy	Proactive intervention adjustment	Predictive analytics	Persistence, engagement, and adaptive functioning

**Table 3:** The inclusion and exclusion criteria according to PICO’s framework.

PICOS Element	Inclusion Criteria	Exclusion Criteria
Population	Individuals with neurodevelopmental disorders or cognitive disorders (mild cognitive impairment)	Neurological and psychiatric disorders
Intervention	AI-powered interventions for therapeutic, educational, or cognitive support	AI used for diagnosis, screening or prediction, non-AI interventions
Comparator	Active control, treatment as usual, waitlist, active and digital controls	Studies without a comparator group
Outcomes	Quantitative measures of cognitive, self-regulatory, and adaptive functioning	Studies reporting only usability, feasibility or technical performance
Study design	Randomized controlled trials (including pilot/feasibility RCTs)	Non-randomized studies, observational studies, qualitative studies, reviews, protocols, conference abstracts, editorials

**Table 4:** The search strings with the main keywords.

The Search Strings with the Main Keywords
“Artificial intelligence” OR “Machine learning” OR “Deep learning” OR “Generative AI” OR “Large language model” OR “Chatbot” OR “Conversational agent” OR “Intelligent tutoring system” OR “Adaptive learning” OR “Virtual agent” OR “Digital therapeutics” OR “Wearable AI” AND “Neurodevelopmental disorder” OR “Attention-Deficit/Hyperactivity Disorder” OR “Autism Spectrum Disorder” OR “Specific Learning Disorder” OR “Dyslexia” OR “Dyscalculia” OR “Intellectual Disability” OR “Communication Disorder” OR “Developmental Coordination Disorder” OR “Cognitive Disorders” OR “Cognitive Impairment” AND “Intervention” OR “Treatment” OR “Therapy” OR “Rehabilitation” OR “Training” OR “Education” OR “Cognitive Training” OR “Behavioural Intervention” OR “Educational Intervention”
AND “Self-regulation” OR “Executive function” OR “Metacognitive skill” OR “Attention regulation” OR “mental flexibility” OR “Emotion regulation” OR “Behaviour regulation” OR “Self-monitoring” OR “Self-control” OR “Working memory” OR “Inhibition control” OR “Social communication” OR “Empathy” OR “Adaptive skills” AND “Randomized Controlled Trial”

**Table 6:** A summary of AI-based intervention effects by skill domain and clinical condition. The neurodevelopmental disorders consist of ADHD, ASD, SLD, ID, and DCD. The category of cognitive disorders is represented in this review by MCI. Each bracketed number refers to one included study. A study reporting outcomes in more than one skill domain is listed once in each relevant domain. For example, a study that assessed both attention and working memory appears in both rows. A study reporting multiple outcomes within the same domain is listed only once for that domain.

Skill Domain	ADHD	ASD	SLD (i.e., Dyslexia and Dyscalculia)	ID	DCD	Cognitive Disorder (Represented in This Review by MCI)
Attention	[57,59,62,63,64,65,67,74,75]					[61,68]
Processing speed	[63]				[76]	
Working memory	[62,64,67,77]		[66]			[61,68]
Metacognitive skills	[57,59]		[73]			
Inhibitory control	[64,65,67,77]				[76]	
Impulse control	[58,65,77]					
Emotional regulation	[58,60,74,75]	[56]				
Mental flexibility	[62,64,77]					[68]
Social skills	[75]	[69,70]				
Language and communication skills	[54,75]	[69,70,71,75]				
Motor control skills	[77]				[76]	
Problem-solving			[66,73]	[55]		
Critical thinking				[55]		
Reading skills	[54]			[72]		

**Table 7:** Summary of effect sizes by skill domain.

Skill Domain	Cohen’s d	η^2^	Hedges’ g	β	Interpretation
Attention	0.28–1.08	0.06–0.56	-	-	Small to large
Processing speed	0.28–0.48	-	-	-	Small to medium
Working memory	0.48–1.42			−0.84 to −1.10	Medium to large
Metacognitive skills	0.56–0.93	0.18–0.58	-	-	Medium to large
Inhibitory control	0.48–0.93	-	0.62	-	Medium to large
Impulse control	0.60–0.83	0.52	-	-	Medium to large
Emotional regulation	0.62–0.83	0.27–0.31	-	-	Medium to large
Mental flexibility	0.63–1.19	0.77	-	-	Medium to large
Social skills	0.62	-	-	-	Moderate
Language and Communication skills	-	0.44–0.90	-	-	Large
Motor control skills	0.48–2.291	-	-	-	Medium to very large
Problem-solving	-	0.10–0.75	-	-	Medium to large
Critical thinking	-	0.749	-	-	Large
Reading skills	-	0.48–0.811	-	-	Large

Note. Cohen’s d, partial eta-squared (η^2^), Hedges’ g, and regression coefficients (β) are not directly comparable and are therefore presented in separate columns. Dashes indicate that the corresponding effect measure was not reported for that domain. The ranges reflect the minimum and maximum values reported across the studies contributing to each domain.

## Data Availability

No new data were created or analyzed in this study. Data sharing is not applicable.

## Supplementary Materials

The following supporting information can be downloaded at https://www.mdpi.com/article/10.3390/healthcare14183102/s1, The Prisma checklist.

## Appendix A

**Table A1:** Characteristics of the included randomized controlled trials. Population labels follow the classification described in Section 3.2. The broad category of neurodevelopmental disorders comprises ADHD, ASD, SLD (specific learning disorder, comprising dyslexia and dyscalculia), DCD (developmental coordination disorder), and MID (mild intellectual disability). The broad category of cognitive disorders is represented in this review by MCI (mild cognitive impairment).

Reference	Country	Intervention Strategy	AI tool	Sample	Clinical Condition	Duration	Measurements	Study’s Design	Skills Developed
Voss et al. [70]	USA	Home-based AI-assisted wearable behavioral intervention combined with standard Applied Behavior Analysis (ABA) therapy	Superpower Glass (Google Glass); wearable AI system using computer vision and machine learning for real-time facial detection and emotion recognition, paired with a smartphone application	Experimental: *n* = 40; Control: *n* = 31; Total: *n* = 71; Mean age = 8.38 ± 2.46 years (6–12 years); Male: 63 (89%); Female: 8 (11%)	ASD	6 weeks (20-min sessions, 4 sessions/week at home, in addition to usual ABA therapy	VABS-II, SRS-2, ABC, CGI, Social Communication Questionnaire, Abbreviated IQ	RCT (parallel-group, waiting-list control)	Emotion recognition, facial engagement, social communication, and adaptive social behavior
Lim et al. [67]	Singapore	BCI-based attention training program	BCI system with machine-learning algorithms that analyze multi-band EEG signals	Total randomized: *n* = 172, (final analysis): *n* = 163 experimental group: *n* = 81, waitlist-control group: *n* = 82, mean age: 8.6 years, male: 147, female: 25	ADHD	8 weeks (24 sessions) and 12 weeks maintenance (3 sessions) 20 weeks total, follow-up at 4 weeks post-intervention	ADHD-RS, CDISC-IV, CBCL, CGAS, CGI-S, CGI-I, PAERS	Single-center, outcome-assessor-blinded, waitlist-controlled, parallel-group RCT	Sustained attention, self-regulation of attention, concentration, academic-task performance (generalization), cognitive engagement, and metacognitive awareness
Kollins et al. [63]	USA	Video game-based digital intervention targeting attentional functioning and cognitive control	AKL-T01 digital therapeutic with adaptive algorithm using staircasing methodology, real-time performance-based difficulty adjustment, and personalized progression	total randomized: *n* = 348, experimental group: *n* = 180, control group: *n* = 168, mean age 9.7, male: 248, female: 100	ADHD	4 weeks (25 min/day, 5 days/week, total 100 sessions)	ADHD-RS-IV, IRS, CGI-I, CGI-S, BRIEF, patient-reported exit questionnaire, parent-reported exit questionnaire	RCT	Attention, cognitive control, interference management, divided attention, selective attention, mental flexibility, response inhibition, processing speed, and sustained attention
Kohn et al. [66]	Germany (collaboration with Switzerland)	Adaptive computer-based mathematics training using an individualized intelligent tutoring approach	Calcularis 2.0—AI-driven adaptive Intelligent Tutoring System that dynamically adjusts task difficulty according to the learner’s performance and learning profile	Experimental: *n* = 34; Control (waiting-list): *n* = 33; Total: *n* = 67; Mean age = 8.96 ± 0.82 years (Grades 2–5); Female: 49; Male: 18	SLD (Dyscalculia)	42 sessions, 20 min/session, over up to 13 weeks	Heidelberg Rechentest (HRT), Number Line Test, Basic Number Processing Computer Test, Mathematics Anxiety Interview (baseline)	RCT	Numerical cognition, arithmetic fluency, number sense, mental number line representation, magnitude comparison, and mathematical problem-solving
Medina et al. [64]	Spain	Home-based AI-driven digital cognitive stimulation program delivered through gamified mobile exercises, compared with a sham video game intervention	AI-driven cognitive training platform using a Case-Based Reasoning algorithm to adapt task type and difficulty according to each child’s cognitive performance	Experimental: *n* = 15; Control: *n* = 14; Total analyzed: *n* = 29 (40 randomized; 11 dropouts); Age: 8–11 years (Experimental: 9.20 ± 1.21; Control: 9.71 ± 1.33 years); Male: 25; Female: 4	ADHD	12 weeks; 3 sessions/week; 15–20 min/session (≥80% adherence required; home-based intervention)	CPT-III, BRIEF, EDAH ADHD Rating Scale, neurophysiological measure: Resting-state Magnetoencephalography (MEG)	Single-center, single-blind, parallel-group RCT	Inhibitory control, sustained attention, working memory, executive functioning, mental flexibility, planning, and cognitive-processing speed
Jang et al. [58]	Republic of Korea	Mobile-based interactive chatbot delivering cognitive behavioral therapy and psychoeducation	Chatbot with AI natural language processing, pre-written dialogue scenarios by psychiatrist, decision tree-based responses, diagrams/pictures as auxiliary tools, emotion-recognition support	*n* = 46 (*n*_exp_ = 23, *n*_clt_ = 23), F = 26, Μ = 20, M_age_ = 25.1	ADHD	4 weeks	CAARS, ASRS, QIDS-SR, SAS, PSS	RCT	Impulse control, emotional regulation, time management, organization skills, and mindfulness
Selaskowski, et al. [59]	Germany	Self-guided digital psychoeducation delivered via an interactive AI chatbot compared with a conventional psychoeducation smartphone application	AI chatbot providing interactive psychoeducation through dialogue, quizzes, and guided-learning modules	Randomized: *n* = 34, experimental = 17, Control = 17, Mean age: 29.6 ± 8.4 years (19–52 years); Female: 18; Male: 16	ADHD	3 weeks	IDA-R, ADHS-SB, WHOQOL, DASS-21, MWT-B	Parallel-group RCT	Self-management, attention regulation, impulsivity management, emotional self-management, coping skills, and symptom awareness
Park et al. [57]	Republic of Korea	Cognitive behavioral therapy (self-instruction training: goal-plan-do-check), behavioral parent training (token-based economies), scaffolding (praise, hint, recruitment)	Voicebot “ForMe” (Raspberry Pi and Google Dialogflow), parent smartphone application	Experimental: *n* = 7, control: *n* = 9, total: *n* = 16, Age: 6–12 years, Mean age: 8.71, Female: 1, Male: 15	ADHD	8 weeks, voicebot used 4 times daily	KGSES, ADHD RS, LPS-C, HSQ PSI-SF, semi-structured interviews	Parallel pilot RCT	Self-regulation, self-efficacy, attention/inattention management, daily task completion (morning routine, homework, organization, and time management), self-control, behavioral problem reduction, and parent–child-relationship improvement
Panda et al. [69]	India	Add-on treatment with AI-based mobile application delivering ABA-based therapy, personalized programs tailored to each child’s needs, discrete trial training, animated avatar guidance, parent-supervised sessions	MPUTE ADT-1: mobile application utilizing face tracking, eye tracking, and body tracking for behavior data collection; reinforcement learning with AI-based algorithms; adaptive-learning algorithm for personalized program evolution; data analytics for progress measurement	Total: *n* = 70, intervention group: *n* = 37, control group: *n* = 33, Age: 2–6 years. Mean age: 3.8 ± 1.6 in intervention; 3.7 ± 1.5 in control) Gender: Predominantly male (30 boys in each group)	ASD	12 weeks (≥60 min/day)	ADOS-2, compliance/adherence, parent feedback questionnaire (10 items), ADI-R, adverse-effects monitoring	Open-label, parallel design, two-arm RCT	Verbal skills, social skills, reduction of repetitive behaviors, receptive language, communication skills, social interaction, and learning engagement
Wang et al. [71]	China	Wearable EEG neurofeedback training targeting the mirror neuron system, powered by machine-learning algorithms	Starkside wearable EEG system (BrainCo) with built-in proprietary machine-learning algorithms, single-channel EEG (Fpz, 160 Hz), and AI algorithms analyze continuous EEG data, classify conditions, and generate mu rhythm probability scores (0–100) every second to control feedback; wearable EEG headband	Total enrolled: *n* = 60, completed (final analysis): *n* = 41, active NFT group: *n* = 17, placebo group: *n* = 24, Age: Mean age 55.0 ± 12.07 months, male: 31, female: 10	ASD	60 sessions (30 min/session, 4–5 days/week, total 12–18 weeks)	CARS-2, ABC, PEP-3, SRS, ATEC	RCT	Expressive language, sensory/cognitive awareness, social reciprocity, affective expression, social communication, sociability, and self-regulation
Aldakhil et al. [75]	Saudi Arabia	AI-based play activities program group format, instructor-led sessions integrating AI-driven activities with hands-on tasks, role-playing, and real-world simulations, parent and teacher involvement through AI tracking systems	Adaptive cognitive exercises, motion-sensing technology, real-time behavior tracking with points-based positive reinforcement, facial-expression recognition, AI-guided meditation	Total: *n* = 61, experimental group: *n* = 30, control group: *n* = 31, age: 8–12 years (M = 10.0, SD = 1.4), female: *n* = 0, male: *n* = 61	ADHD	4 weeks	PedsQL		Attention control, behavioral regulation, social interaction, emotional regulation, cognitive skills (problem-solving, memory, and reasoning), motor skills, mindfulness, self-regulation, focus, teamwork, and communication
Xu et al. [74]	China	AI-assisted drawing therapy vs. Traditional drawing therapy (control), based on the Hooked Behavior Model (Trigger, Action, Variable Reward, Investment), with phased thematic design (self-awareness, emotions, past experiences)	Midjourney Vision 5 (AI image generation based on children’s drawings and therapist-coded keywords)	Total: *n* = 41, Experimental group: *n* = 19, Control group: *n* = 22, Age: 7–10 years, Female: *n* = 7, Male: *n* = 34	ADHD	24 weeks (6 months) between baseline and post-intervention, 24 weekly sessions (20–30 min each)	SNAP-IV-26, WFIRS-P	Parallel RCT	Attention/inattention reduction, hyperactivity–impulsivity reduction, oppositional defiant behavior reduction, functional improvements (family, school, life skills), self-concept enhancement, emotional regulation, and self-expression
Koegel et al. [56]	USA	AI-based empathy training program using Large Language Model (GPT-4) with live feedback, sentiment rating (positive/neutral/negative), graded verbal responses, verified sample answers, non-open domain chatbot	Noora (AI chatbot using GPT-4 via Microsoft Azure OpenAI Services), web app accessible via computer, tablet, or phone	Total: *n* = 30, Experimental group: *n* = 15, Control (waitlist) group: *n* = 15, Mean: 18.3, Female: *n* = 4, Male: *n* = 26	ASD	4 weeks (10 trials/day, 5 days/week, expected total ~200 trials)	Waitlist-controlled RCT		Verbal empathetic responses, sentiment recognition (positive/neutral/negative), responding to others’ fortune/misfortune, showing understanding and concern, asking relevant questions, and social conversation skills
Bilan et al. [65]	Spain	AI-driven Digital Cognitive Therapy (serious game) delivered via mobile device (tablet/smartphone), personalized and adaptive cognitive training with AI algorithms adjusting task selection and difficulty based on individual performance	Sincrolab DCT (KAD_SCL_01)—AI-powered digital cognitive program with 14 cognitive game-based tasks (go/no-go and n-back), adaptive algorithms for personalized intervention	Total: *n* = 41 Experimental group: *n* = 20 Control (sham) group: *n* = 21 Age: 8–12 years (Mean: 9.41 experimental, 9.38 control) Female: *n* = 3 (7.3%) Male: *n* = 38 (92.7%)	ADHD	12 weeks (3 sessions/week, 15 min/session, total 36 sessions)	CPT-3, NEPSY-II, EDAH, BRIEF, WNV	Single-center, parallel, single-blindRCT	Inhibitory control/impulsivity reduction, inattention reduction, sustained attention, vigilance, working memory, mental flexibility, spatial processing, response inhibition, and attention regulation
Ferizaj et al. [61]	Germany	Mobile, gamified computerized cognitive training using the NeuroNation MED app (self-administered, multi-domain cognitive training)	NeuroNation MED	Total: *n* = 50, Intervention group: *n* = 36 Control group (waiting): *n* = 14 Age: Mean 58.1 years Female: 36, Male: 14	MCI	12 weeks	S-NAB, CFQ-D, HADS-D, HLQ-D, TICS	Multicenter, parallel RCT	Planning and problem-solving, verbal fluency, mental flexibility, inhibitory control, and working memory
Dong et al. [54]	China	AI chatbot-led shared book reading using PEER (Prompt, Evaluate, Expand, and Repeat) and CROWD (Completion, Recall, Open-ended, Wh-questions, and Distancing) dialogic-reading techniques	ChatGPT 4.0 (AI chatbot with oral input and voice output, trained with predefined instructions, emotion-neutral feedback, programmable repetitions, and dynamic pacing adjustment via NLP)	Total: *n* = 153 chatbot: *n* = 40, Mean age = 68.13 months	ADHD	12 weeks (2 sessions/week, 25 min/session)	RV, EV, CR, LC, RI, RAX	RCT with 4 groups	Receptive vocabulary, expressive vocabulary, character reading, listening comprehension, syntax (sentence-structure awareness), reading interest, and reading-anxiety reduction
Ofem et al. [55]	Nigeria	AI-scaffolded planning tools intervention, step-by-step prompts, examples, and constructive feedback, structured activities progressively enhancing research topic formulation skills, adaptive prompts and personalized feedback	ChatGPT (brainstorming, idea generation, and topic clarification), Elicit (literature exploration, feasibility analysis, evidence-based refinement), Research Rabbit (mapping connections between research ideas; strengthening alignment with research objectives)	Total: *n* = 91, Experimental group: *n* = 46, control group: *n* = 45, Age: School-aged (third-year students) Female: 41 Male: 50	MID	8 weeks	RTFS	RCT	Critical thinking, problem-solving, and academic self-efficacy
Alsolami et al. [72]	Saudi Arabia	AI-based personalized academic skills training, 1-on-1 instruction with teacher, adaptive learning, gamification, interactive apps, multimedia learning, visual aids, positive reinforcement, graduated guidance	Multiple AI-powered educational applications	Total: *n* = 70, experimental group: *n* = 35, control group: *n* = 35 Mean age = 10.5 Female: 0 Male: 70	MID	5 weeks (10 sessions, 2 sessions/week, 60 min each)	WJ-IV-ACH, WISC-V, VABS-2	RCT	Phonemic/phonological awareness, word recognition/decoding, reading fluency, and reading comprehension
Zhu et al. [77]	China	mHealth-facilitated personalized exercise rehabilitation program, merging physically and cognitively demanding exercises	mHealth system that uses AI algorithms to recommend personalized exercises based on each participant’s assessment results, dynamic adaptation of programs based on individual progress and preferences, automated tracking and feedback, reward mechanisms	Total: *n* = 144, experimental group: *n* = 53, traditional offline group: *n* = 45, control group: *n* = 46, mean age 8.53, male: 126, female: 18	ADHD	12 weeks (3 sessions/week, 45–60 min/session, total 36 sessions)	TGMD-3, MABC-2, SNAP-IV	Single-center, assessor-blinded, parallel, three-arm RCT	Inhibitory control, impulse control, working memory, mental flexibility, and motor skills (gross motor, fine motor, and balance),
Demirci et al. [76]	Turkey	AI-supported occupational therapy program based on the Model of Human Occupation	Multiple AI applications: digital coloring, dot-to-dot activities, machine-learning tools for shadow figure and finger movement analysis in real-time, deep learning models for pencil grip and drawing speed assessment, AI-assisted handwriting analysis with real-time feedback and error correction	Total: *n* = 42, Intervention group: *n* = 21, control group: *n* = 21, Mean age = 114.33 ± 12.75 months, male: *n* = 25, female: *n* = 17	DCD	8 weeks (2 sessions/week, 45 min/session, total 16 sessions)	MHA, DCDQ, BOT2-BF, MVPT-3	RCT	Handwriting skills (writing speed, legibility, shape, alignment, size, and spacing), fine motor control, visual–motor integration, motor planning, spatial awareness, hand–eye coordination, motor learning, and grip and stroke control
Bergmann et al. [62]	Germany	Individualized digital cognitive training using the NeuroNation MED app	NeuroNation MED (digital cognitive training application)	Initially enrolled: *n* = 77 Final randomized comparison: *n* = 60, training condition datasets: *n* = 28, TAU condition: *n* = 32, Age: Mean age = 33.4, Male = 16, Female = 25, Diverse = 2	ADHD	12 weeks	JFSKB-II, CAARS-L SB, WHOQOL-BREF, WFIRS Scale, BDI-II, BFI, SUS, WURS-K, TAP, VLMT, RWT, WST	Single-blind RCT with cross-over design	Mental flexibility, response inhibition, working memory, processing speed attention (sustained and divided), memory, reasoning, and verbal fluency
Kim et al., 2026 [68]	Republic of Korea	AI-driven, self-guided, home-based cognitive telerehabilitation, autonomous difficulty adjustment via AI algorithm, cloud-based compliance monitoring, personalized content recommendations, no direct therapist supervision, tablet-based delivery	AI-driven computerized cognitive rehabilitation platform with: training record module, task analysis module, performance prediction module, recommendation module, adaptive difficulty algorithms, cloud-based infrastructure	Total: *n*= 70, Final analyzed: *n* = 62 Group AB (Intervention-first): *n* = 33 Group BA (Control-first): *n* = 29 Age: Mean 74.0 ± 4.8 years Female: 54, Male: 8	MCI	5 weeks (24 sessions)	K-MMSE2, DSF, DSB, TMT-A, TMT-B, K-CESD-R, EQ-5D, SES, S-IADL	RCT	Mental flexibility, inhibitory control, planning, organization, problem-solving, task switching, attention, and working memory
Wang et al. [73]	Taiwan	AI chatbot-assisted learning for arithmetic word-problem-solving, adaptive, multisensory, and individually tailored support, stepwise scaffolded problem-solving, graduated prompt hierarchy, text-to-speech functionality, color-coded keyword highlighting	GPT-based AI Chatbot configured with a curated corpus of 150 curriculum-aligned arithmetic word problems, fine-tuned with stepwise solution paths, scaffolded feedback, immediate hints, adaptive difficulty adjustment, text-to-speech integration	Total: *n* = 83, experimental group: *n* = 39, control group: *n* = 44 Mean age = 10.52, Male: 44, Female: 39	SLD (Dyslexia)	6 weeks (2 sessions/week, 30 min/session, total 12 sessions)	AWP, AMS	RCT	Arithmetic word-problem-solving, problem representation, operation selection, equation/number sentence construction, computation, checking and reasoning, mathematical reasoning, and reading comprehension in mathematical contexts
Miao et al. [60]	China	Parent–child storybook reading intervention (dialogic reading vs. typical reading) with parent training via AI chatbot or professional social workers	AI chatbot, audio input, automatic conversation transcription, immediate feedback, personalized training at parent’s own pace	Total: *n* = 249, Experimental group: 198, control group: 51 Mean age = 5.76, Total Male: 159, Female: 90	ADHD-PI and ADHD-C	4 weeks (2 sessions/week, 25 min/session)	PreBERS IEB, UERS-Q, UERS-C	RCT	Emotional regulation, school readiness, social confidence, reduction of impulsive emotional behavior, and increased use of emotional-regulation strategies

RCT: randomized controlled trial, ASD: autism spectrum disorder, ADHD: attention-deficit/hyperactivity disorder, VABS-II: Vineland Adaptive Behavior Scales-II, SRS-2: Social Responsiveness Scale-2, ABC: Aberrant Behavior Checklist, CGI: Clinical Global Impression, IDA-R: Integrated Diagnosis of ADHD in Adulthood–Revised, ADHS-SB: ADHD Self-Assessment Scale World, WHOQOL: Health Organization Quality of Life Questionnaire, DASS-21: Depression–Anxiety–Stress Scales, MWT-B: Multiple-Choice Word Test, KGSES: Korean General Self-Efficacy Scale, ADHD RS: ADHD Rating Scale, LPS-C: Life Participation Scale for ADHD Children, HSQ: Home Situation Questionnaire, PSI-SF: Parenting Stress Index Short-Form, SNAP-IV-26: Swanson, Nolan, and Pelham—IV Rating Scales (26 items), WFIRS-P: Weiss Functional Impairment Rating Scales—Parent Form, ERAP: Empathetic Response Rate Within the AI Program, HCS: Human Conversational Sample, CSQ: Confidence Survey Questionnaire, ASQ: Acceptability/Satisfaction Questionnaire, CPT-3: Conners Continuous Performance Test 3rd Edition, NEPSY-II: Developmental Neuropsychological Assessment II, EDAH: Scale for Evaluation of ADHD, BRIEF: Behavior Rating Inventory of Executive Function, WNV: Wechsler Non-Verbal Scales, S-NAB: Neuropsychological Assessment Battery Screening Module, CFQ-D: Cognitive Failure Questionnaire—German version, HADS-D: Hospital Anxiety and Depression Scale—German version, HLQ-D: Health Literacy Questionnaire—German version, TICS: Telephone Interview for Cognitive Status, JFSKB-II: Jülich Questionnaire on Subjective Cognitive Decline, CAARS-L SB: Conners’ Adult ADHD Rating Scales, WHOQOL-BREF: World Health Organization Quality of Life, WFIRS: Weiss Functional Impairment Rating Scale, BDI-II: Revised Beck’s Depression Inventory, BFI: Brief Fatigue Inventory, SUS: System Usability Scale, WURS-K: Wender Utah Rating Scale, TAP: Test of Attentional Performance, VLMT: Verbal Learning and Retention Test, RWT: Regensburg Verbal Fluency Test, WST: Vocabulary Test, PedsQL: Pediatric Quality of Life Inventory, RV: Receptive Vocabulary, EV: Expressive Vocabulary, CR: Character Reading, LC: Listening Comprehension, RI: Reading Interest Questionnaire, RAX: Reading Anxiety Questionnaire, CAARS: Conner’s Adult ADHD Rating Scales, ASRS: Adult ADHD Self-Report Scale v1.1, QIDS-SR: Quick Inventory of Depressive Symptomatology—Self-Report version, SAS: Self-rating Anxiety Scale, PSS: Perceived Stress Scale, WJ-IV-ACH: Woodcock-Johnson IV Tests of Achievement, WISC-V: Wechsler Intelligence Scale for Children—Fifth Edition, VABS-2: Vineland Adaptive Behavior Scales—Second Edition, RTFS: Research Topic Formulation Scale, MID: Mild Intellectual Disabilities, AI-SPT: Artificial Intelligence-Scaffolded Planning Tools, IRB: Institutional Review Board, ANCOVA: Analysis of Covariance, I-CVI: Item Content Validity Index, S-CVI: Scale Content Validity Index, K-MMSE2: Korean Mini-Mental State Examination 2nd Edition, DSF: Digit Span Forward, DSB: Digit Span Backward, TMT-A: Trail Making Test Part A, TMT-B: Trail Making Test Part B, K-CESD-R: Korean Center for Epidemiological Studies Depression Scale—Revised, EQ-5D: EuroQoL 5-Dimension, SES: Self-Efficacy Scale, S-IADL: Seoul-Instrumental Activities of Daily Living, CCI: Charlson Comorbidity Index, ADOS-2: Autism Diagnostic Observation Schedule—2nd Edition, ADI-R: Autism Diagnostic Interview—Revised, PreBERS: Preschool Behavioral and Emotional Rating Scale, UERS-Q: Use of Emotional Regulation Strategies—Questions, UERS-C: Use of Emotional Regulation Strategies—Cooperative Adjustment, AWP: Arithmetic Word-Problem-Solving Task, AMS: Academic Motivation Scale, DCD: Developmental Coordination Disorder, MHA: Minnesota Handwriting Assessment, DCDQ: Developmental Coordination Disorder Questionnaire, BOT2-BF: Bruininks–Oseretsky Test of Motor Proficiency 2 Brief Form, MVPT-3: Motor-Free Visual Perception Test-3, CARS-2: Childhood Autism Rating Scale Second Edition, ABC: Autism Behavior Checklist, PEP-3: Psychoeducational Profile Third Edition, SRS: Social Responsiveness Scale, ATEC: Autism Treatment Evaluation Checklist, ADHD-RS: ADHD Rating Scale, CDISC-IV: Computerized Diagnostic Interview Schedule for Children Version IV, CBCL: Child Behavior Checklist, CGAS: Children’s Global Assessment Scale, CGI-S: Clinical Global Impression—Severity, CGI-I: Clinical Global Impression—Improvement, PAERS: Pediatric Adverse Events Rating Scale, DHD-RS-IV: ADHD Rating Scale IV, IRS: Impairment Rating Scale, BRIEF: Behavior Rating Inventory of Executive Function, MINI-KID: Mini International Neuropsychiatric Interview for Children and Adolescents, API: Attention Performance Index, TGMD-3: Test of Gross Motor Development 3rd Edition, MABC-2: Movement Assessment Battery for Children 2nd Edition, SNAP-IV: Swanson, Nolan, and Pelham version IV rating scale, RTFS: Research Topic Formulation Scale.
